# Early nervous system development in the chaetognath *Spadella cephaloptera* exhibits conserved bilaterian patterning features

**DOI:** 10.3389/fcell.2026.1819435

**Published:** 2026-05-28

**Authors:** June F. Ordoñez, Alice Frisinghelli, Cristian Camilo Barrera Grijalba, Tim Wollesen

**Affiliations:** 1 Unit for Integrative Zoology, Department of Evolutionary Biology, University of Vienna, Vienna, Austria; 2 Vienna Doctoral School of Ecology and Evolution (VDSEE), University of Vienna, Vienna, Austria

**Keywords:** gene expression, neurogenesis, dorsoventral patterning, central nervous system, Spiralia, Gnathifera

## Abstract

Nervous systems display extensive diversity in structure and organization, yet a broadly conserved set of signaling pathway components and transcription factors is consistently associated with early neurogenesis in many animal lineages. Determining how these conserved markers map onto the spatiotemporal organization of neurogenic territories across phylogenetically informative but underrepresented lineages, particularly within Spiralia, is critical for refining inferences about the evolutionary origins and diversification of nervous systems. Chaetognaths, a spiralian lineage frequently recovered close to Gnathifera, have a compact and centralized nervous system but lack detailed molecular descriptions of early neural development. Here, we generate an expression-based developmental map of early neurogenesis in the chaetognath *Spadella cephaloptera* by combining nuclear-staining–based anatomical staging with spatiotemporal analyses of conserved developmental genes associated with early neurogenesis and axial patterning from gastrulation through early post-embryonic stages. *Sce-soxB1-like1* and *Sce-neuroD* expressions mark a lateral neuroectodermal territory during gastrulation. Notably, *Sce-neuroD* is activated early in a broad ectodermal domain and is expressed within mitotically active neuroectodermal cells, consistent with early deployment in proliferative neurogenic territories. *Sce-soxB1* and *Sce-soxB2* show delayed and more spatially restricted expression relative to *Sce-soxB1-like1*, suggesting a paralog-specific partitioning of SoxB deployment during chaetognath neurogenesis. *Sce-bmp2/4* and *Sce-chd* exhibit reciprocal dorsoventral expression during gastrulation that coincides with early neurogenic territory formation, before transitioning to more localized expression later in development. *Sce-nk6* and *Sce-hb9* reveal early ventral regionalization of the developing ventral nerve center (VNC), with *Sce-hb9* occupying a subset of a broader *Sce-nk6* domain, in line with conserved ventral subtype-associated regionalization. *Sce-th* (*tyrosine hydroxylase*) is detected in a small bilateral subset of hatchling VNC cells, while *Sce-dbh* (*dopamine beta-hydroxylase*) is first detected only in early juveniles in the anterior VNC and head domains, suggesting stage-dependent and region-specific deployment of catecholamine-pathway components. Together, these expression-based datasets provide a comparative reference for early neurogenesis in chaetognaths and a framework for assessing conserved and lineage-specific features of early neurogenic patterning across Spiralia.

## Introduction

Nervous systems exhibit remarkable diversity in organization, ranging from basiepithelial nerve nets of cnidarians to the highly centralized brains and nerve cords of many bilaterians (Holland, 2003; Schmidt-Rhaesa, 2007). Despite this architectural divergence, comparative developmental studies have consistently identified a shared repertoire of signaling pathways and transcription factors associated with early neural development (Arendt et al., 2016; 2008; De Robertis and Sasai, 1996; Hartenstein and Stollewerk, 2015). In well-studied model systems, signaling pathways (e.g., Bone Morphogenetic Protein (BMP)/Chordin signaling) have been associated with the positioning of neurogenic territories, while transcription factors, most prominently members of the Sox and basic helix–loop–helix (bHLH) families, are widely implicated in neural competence, patterning, and neuronal differentiation (Bertrand et al., 2002; Guth and Wegner, 2008; Kelava et al., 2015; Mizutani and Bier, 2008; Quan and Hassan, 2005; Sprecher and Reichert, 2003; Vervoort and Ledent, 2001). The recurrent involvement of these components across distantly related taxa suggests that early neurogenesis involves deeply conserved molecular signatures (Arendt et al., 2016; Faltine-Gonzalez et al., 2023). However, whether similarities in gene expression correspond to comparable spatial organization of early neurogenic events across lineages remains unclear.

Expanding molecular studies beyond traditional model organisms has begun to refine this picture. Conserved neural-associated transcription factors are widely reported across major bilaterian clades, including acoels, ecdysozoans, deuterostomes, and spiralians (Denes et al., 2007; Janssen et al., 2018; Lowe et al., 2003; Martín-Durán et al., 2018; Martinez et al., 2024). Within Spiralia, studies in annelids, mollusks, and nemerteans demonstrate that many of these genes are expressed during early neurogenesis, yet their timing, spatial relationships, and cellular contexts vary among taxa, and in some cases, even between closely related species (Buresi et al., 2016; Deryckere et al., 2021; Monjo and Romero, 2015a; Simionato et al., 2008; Sur et al., 2020). Such variation underscores that shared molecular markers can be associated with distinct spatiotemporal configurations of neural development. However, available spiralian developmental datasets remain heavily skewed toward lophotrochozoan model taxa, whereas comparable data from Gnathifera, a major spiralian clade sister to Lophotrochozoa, are scarce. This uneven taxonomic sampling constrains assessments of how broadly patterns inferred from lophotrochozoan models generalize across Spiralia and consequently limits broader inferences about conserved features of bilaterian neurogenesis. Moreover, distinguishing deep homology from convergence in nervous system evolution ultimately requires dense comparative sampling, and expression-based maps from understudied spiralian lineages provide an essential baseline (Hejnol and Lowe, 2015).

Chaetognaths (arrow worms) are marine invertebrates frequently recovered as closely affiliated with Gnathifera, based on recent phylogenomic analyses (Laumer et al., 2019; Marlétaz et al., 2019). The adult nervous system has a compact, non-segmented organization, with an anterior cerebral ganglion connected via circumesophageal connectives to an intraepidermal ventral nerve center (VNC) in the trunk (Goto and Yoshida, 1987; Harzsch et al., 2009; Harzsch and Müller, 2007; Rieger et al., 2010). Classical embryological studies already described key anatomical features of chaetognath neurodevelopment, including a two-layered gastrula organization and the ectodermal origin of the VNC (Doncaster, 1902; John, 1933). In the benthic chaetognath *Spadella cephaloptera*, histological observations showed that the lateral ectoderm thickens during gastrulation, after which ectodermal nuclei divide and shift inward to form the ganglion cell masses of the VNC (John, 1933). During elongation, comparable thickening of the lateral cephalic ectoderm gives rise to the cerebral ganglia, while the trunk ganglion cells become fully separated from the overlying ectoderm. Subsequent works using immunohistochemical approaches have characterized neuronal subtypes and documented the progressive maturation of neural structures during post-embryonic development (Goto et al., 1992; Perez et al., 2013; Rieger et al., 2011). However, the molecular underpinnings of early neurogenesis in chaetognaths, including the spatial and temporal deployment of genes consistently associated with early nervous system development across diverse lineages, remain largely unexplored, limiting direct comparisons with other spiralians.

In this study, we provide an expression-based developmental map of early neurogenesis in the chaetognath *S. cephaloptera* by integrating nuclear staining-based anatomical characterization with spatiotemporal analysis of broadly conserved neural-associated genes (including SoxB/bHLH factors such as *soxB1* and *neuroD*, dorsoventral (DV) patterning genes *bmp2/4* and *chordin*, and early subtype-associated markers *nk6* and *hb9*). By tracing gene expression dynamics from gastrulation through early post-embryonic stages, we delineate the spatial and temporal organization of early neurogenic events in this lineage. Together, these data offer an independent comparative reference for evaluating how these markers associated with nervous system development are deployed across Spiralia.

## Methods

### Specimen collection and fixation

Field sampling, animal husbandry, developmental staging, fixation and storage procedures were described in (Ordoñez and Wollesen, 2025). Briefly, adult *S. cephaloptera* were collected from the intertidal zone of Roscoff, France, and maintained in aquaria at 14 °C in 35‰ natural seawater at the University of Vienna. Embryos and post-hatching stages (hatchling and early juveniles) were fixed in 4% paraformaldehyde (PFA) prepared in either MOPS buffer or filtered natural seawater, following the buffer composition and fixation times optimized for ISH and HCR in (Ordoñez and Wollesen, 2025). Specimens were washed with PBST (PBS containing 0.1% Tween-20) and stored in methanol at −20 °C until use.

### Candidate gene identification and orthology assessment

Putative homologs of candidate genes were identified from the *S*. *cephaloptera* draft transcriptome (Wollesen et al., 2023a) through blastx (Altschul et al., 1990) comparisons with sequences from the NCBI non-redundant (nr) protein database. We targeted regulatory genes representing complementary stages of neurogenesis: (i) factors associated with neural competence and early neuroectodermal specification (*soxB1*), (ii) regulators commonly implicated in neuronal differentiation or commitment (*soxB2* and the proneural bHLH *neuroD*), (iii) DV signaling components (*bmp2/4*, *chordin* (*chd*)), and (iv) ventral patterning and motor-program homeobox genes (*nk6*, *hb9*), along with enzymes involved in catecholaminergic neurotransmitter biosynthesis (*dopamine beta-hydroxylase, dbh*; *tyrosine hydroxylase*, *th*).

Protein translations were aligned with MAFFT v7.490 (Katoh, 2005; 2002) implemented in Geneious Prime (v2023.0.1), and trimmed with ClipKit v2.3.3 (Steenwyk et al., 2020) followed by manual curation. Orthology was assessed with maximum-likelihood (ML) analysis in IQ-TREE v2, employing ModelFinder for site-heterogeneous model selection and 1,000 ultrafast bootstrap and SH-aLRT replicates (Hoang et al., 2018; Kalyaanamoorthy et al., 2017; Minh et al., 2020). Resulting phylogenetic trees were visualized using the native tree viewer in Geneious Prime. Reference amino acid sequences used in each phylogenetic analysis are listed in Supplementary Table S1. MAFFT alignment settings, the final retained alignment region, and the evolutionary model selected for each analysis are provided in Supplementary Table S2.

### Whole-mount fluorescent *in situ* hybridization (FISH) and imaging

Gene expression was examined using hybridization chain reaction (HCR-FISH) (Choi et al., 2014; 2018) for all markers except *dbh* and *th*. Experiments followed the protocol of Choi et al. (2018) as modified by Bruce et al. (2021), with additional adjustments from Ordoñez and Wollesen (2025). Specific HCR probe sets were designed using *insitu_probe_generator.py* (https://github.com/rwnull/insitu_probe_generator (Kuehn et al., 2022)) and were synthesized by Integrated DNA Technologies (München, Germany). For *Sce-bmp2/4*, probe concentration was increased threefold because the short transcript length only produced nine pairs of probes. Negative controls were performed using hairpins only, without probe incubation, and were imaged with the same hairpin/fluorophore combinations and confocal settings used for the corresponding experimental samples.

Due to limited embryo availability, mid-gastrula stage was not assayed for *Sce-soxB1*, *Sce-soxB2*, and selected double-labeling (*Sce-soxB1-lik*e*1*/*neuroD*, *Sce-chd*/*neuroD*, and *Sce-nk6*/*hb9*). Likewise, hairpin-only negative controls were performed only for late gastrula, early elongation, and hatchlings, because embryo availability at the early and mid-gastrula stages was very limited.

Expression of *dbh* and *th* was detected using traditional chromogenic/fluorescent ISH approach following Ordoñez and Wollesen (2024) and Wollesen et al. (2023b) for riboprobe synthesis, digoxigenin-labeling, and hybridization. These assays were restricted to hatchlings and early juveniles, since the current protocol does not yield reliable signal in embryos (Ordoñez and Wollesen, 2025). All riboprobes, HCR probe sets, and amplifiers are listed in Supplementary Tables S3, S4.

Images were acquired using Leica TCS SP5 confocal laser scanning microscope (Leica Microsystems, Heidelberg, Germany). All images were processed in Fiji (Schindelin et al., 2012) and Figures were assembled using Inkscape (https://inkscape.org).

## Results

### Orthology assignment of candidate neural regulators

We recovered putative single-copy orthologs of *soxB1*, *soxB2*, *neuroD, bmp2/4*, *chordin*, *nk6*, *hb9*, *th*, and *dbh* from the *S. cephaloptera* draft transcriptome. Maximum-likelihood phylogenetic analyses confirmed their identity, with each gene clustering with the corresponding bilaterian orthologs (Supplementary Figures S1–S8).

Aside from *Sce-soxB1*, we identified two highly similar SoxB1-like sequences from the transcriptome, both initially identified as *soxB1* candidates in blastp searches against the nr database. *Sce-soxB1-like1* showed the highest similarity to Sox2-like from *Tubulanus polymorphus* (e-value 2e^−46^; XP_074658127.1), whereas *Sce-soxB1-like2* matched Sox2 from *Trichuris trichiura* (e-value 8e^−44^; CDW55571.1). Sce*-soxB1-like2* is 5′-partial relative to the complete *Sce-soxB1-like1* ORF and shares only 33.83% nucleotide identity across the aligned shared coding region (Supplementary Data). Although the HMG box is highly conserved between the two sequences (85.9% amino acid identity), pairwise comparison of the aligned shared protein region using MAFFT shows only 29.17% amino acid identity (Supplementary Figure S9; Supplementary Data). In the ML phylogeny, the two sequences formed a strongly supported cluster (SH-aLRT = 98.7%; ultrafast bootstrap support = 100%; Supplementary Figure S7). Together, these observations argue against their interpretation as transcriptome assembly artifacts of the same gene and instead support their treatment as distinct SoxB1-like transcripts, consistent with the presence of putative SoxB1-like paralogs in *S. cephaloptera*. Because *soxB1-like1* is the longer and more complete transcript, we used this sequence for HCR probe design to characterize *soxB1-like* expression.

### Anatomical organization of the developing nervous system in embryos of *Spadella cephaloptera*

**FIGURE 1:**
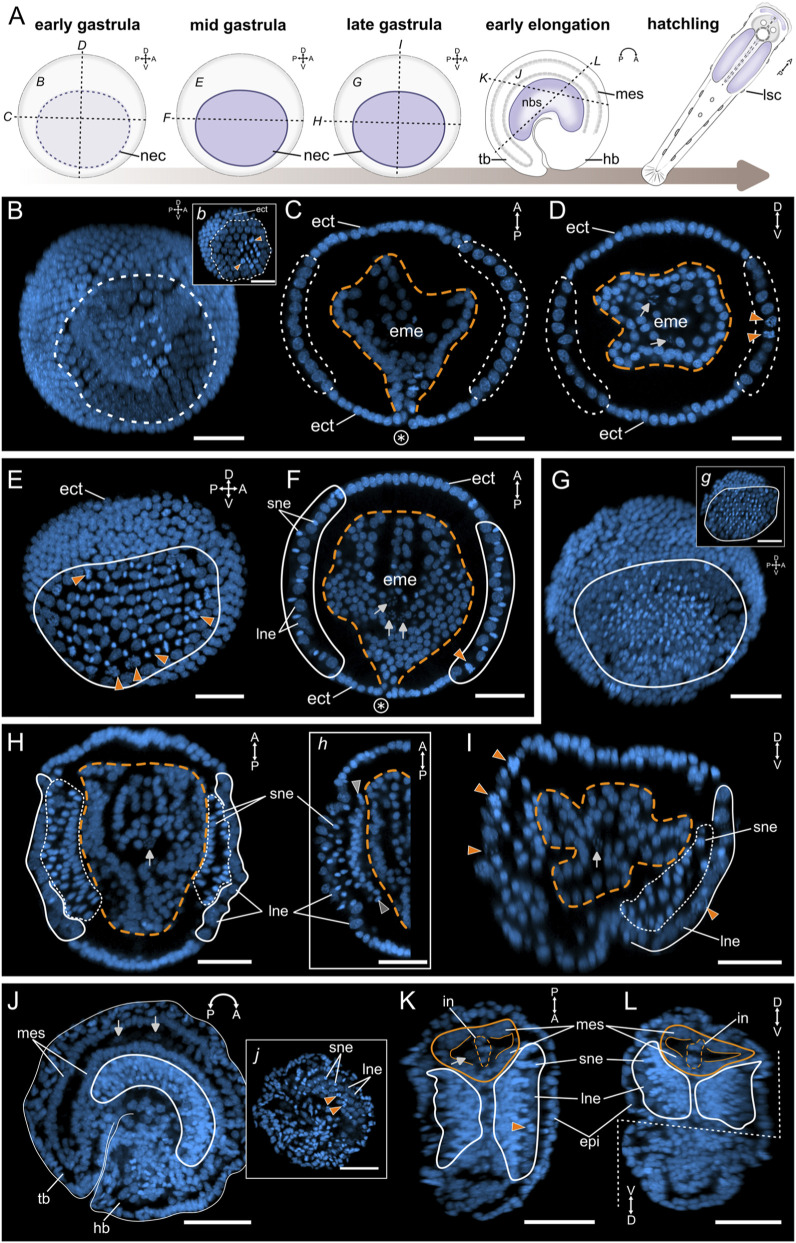
DAPI-based anatomical characterization of ventral nervous system development in *Spadella cephaloptera* embryos. **(A)** Schematic overview of *S. cephaloptera* development from gastrulation to hatching (early, mid, and late gastrula; early elongation; hatchling); embryos are shown in lateral view and the hatchling in dorsal view. Neurogenic regions, including the neuroectoderm (NEC) and neural cells of the developing ventral nerve cord (VNC), are indicated in purple. At the early gastrula stage, the purple domain corresponds to the presumptive neuroectoderm. Labeled dotted lines indicate the plane along which the embryo was optically sectioned in the corresponding confocal panels. **(B–D)** Early gastrula. **(B)** Lateral maximum projection. Inset b highlights a region of the outer ectoderm showing comparatively large, DAPI-faint nuclei within the presumptive neuroectoderm, contrasted with surrounding non-neural ectodermal nuclei. **(C,D)** Dorsal **(C)** and transverse **(D)** sections. **(E,F)** Mid gastrula. **(E)** Lateral maximum projection. **(F)** Dorsal section. **(G–I)** Late gastrula. **(G)** Lateral maximum projection. Inset *g* shows the surface ectoderm highlighting large-nucleus neuroectodermal (LNE) cells and small-nucleus neuroectodermal (SNE) cells within the NEC, as well as surrounding non-neural ectoderm. **(H,I)** Dorsal **(H)** and transverse **(I)** sections showing an outer layer of LNE cells (solid white outline), an inner layer of SNE cells (dotted white outline), and underlying mesodermal and endodermal tissues (dashed orange outline). Inset *h* highlights the compact inner band of SNE cells at the ectoderm–endomesoderm interface (gray arrowheads). **(J–L)** Early elongation. **(J)** Lateral maximum projection. Inset *j* highlights LNE and SNE cells within the nascent ventral nerve cord. **(K, L)** Dorsal **(K)** and transverse **(L)** sections showing the nascent VNC, with the outer LNE layer appearing to be positioned subepidermally. Graphic annotations: White outlines indicate neural territories; orange outlines indicate the endomesoderm in gastrula stages and mesodermal cells and the intestinal region in the early elongation stage; orange arrowheads indicate morphologically identifiable mitotic figures; gray arrows indicate primordial germ cells; an encircled asterisk indicates the blastoporal region. Scale bars: 50 μm. Orientation is indicated in the upper right corner of each panel. Abbreviations: ect, ectoderm; eme, endomesoderm; epi, epidermis; hb, head bud; in, intestine; lne, large-nucleus neuroectodermal cells; mes, mesodermal cells; nbs, neural cells of the developing VNC; nec, neuroectoderm; sne, small-nucleus neuroectodermal cells; tb, tail bud.

Classical histological work on *S. cephaloptera* early developmental stages provided the earliest description of its general embryonic territories and cellular organization (John, 1933). Although these observations did not identify neural progenitors or define neurogenic mechanisms (in contemporary terms), they established the basic epithelial architecture in regions that later give rise to neural tissues, such as the brain and VNC. These foundational observations form the basis for our descriptive assessment of early neuroectodermal organization. Here, we complement earlier work by detailing the spatial arrangement and nuclear characteristics (e.g., nuclear shape and DAPI signal intensity) of the presumptive neuroectoderm across the early gastrula, mid-gastrula, late gastrula, and early elongation stages (Figure 1).

During early gastrula, the ectoderm contains a bilateral lateral field of comparatively larger, relatively DAPI-faint nuclei, distinguishable from adjacent epithelia by size (Figures 1A–D). We refer to this field as the presumptive neuroectoderm (NEC), based mainly on its position and its later co-localization with *Sce-neuroD* and *Sce-soxB1-like1* domains described below.

By mid-gastrula, the NEC becomes morphologically recognizable as nuclei within the ventrolateral ectoderm show pronounced size heterogeneity (Figures 1E,F). Cells with large, DAPI-faint nuclei and cells with small, DAPI-bright, and teardrop/ovoid nuclei (visible in transverse and dorsal views) occur in proximity and often alternate along short stretches. For clarity and consistency, we refer to large-nucleus neuroectodermal cells as “LNE cells” and small-nucleus neuroectodermal cells as “SNE cells” throughout the remainder of this manuscript. From mid-gastrula through early elongation, LNE cells within this candidate NEC show frequent mitotic figures (orange arrowheads in Figure 1), supporting the interpretation that this domain includes a proliferative neuroectodermal population (putative progenitor-like cells).

In the late gastrula, the SNE cells appear to shift basally, populating the ventrolateral margins of the endomesoderm as tightly packed clusters (Figures 1G–I). Consequently, the ventrolateral ectoderm appears stratified: (i) a surface layer of LNE cells along the outer ectodermal epithelium, (ii) an intermediate subepidermal layer of subepidermal layer of SNE cells that appear to have shifted inward, and (iii) an inner band of SNE cells at the ectoderm–endomesoderm interface (between the two gray arrowheads in inset *h* of Figure 1H).

At early elongation, when the embryo already exhibits pronounced C-shaped axial curvature, the inner SNE-cell layer thickens and resolves into bilateral longitudinal bands that converge medioventrally to form the nascent VNC (Figures 1J–L). LNE cells now appear as a subepithelial layer separated from the epidermis and positioned at the outer margin of the SNE-derived VNC (Figures 1K,L). In the head bud, John (1933) described a thickened lateral cephalic ectoderm with nuclei positioned nearer the underlying mesodermal masses, interpreted as prospective cerebral ganglion cell precursors. However, in our material, this primordium cannot be delineated with confidence from DAPI morphology alone.

By itself, this nuclear architecture does not define progenitors or precursors, but the staging and terminology (e.g., LNE/SNE cells) described here provide the positional framework used in the subsequent sections to map and interpret gene-expression domains.

### Expression of *soxB* paralogs and *neuroD*

#### Sce-soxB1

**FIGURE 2:**
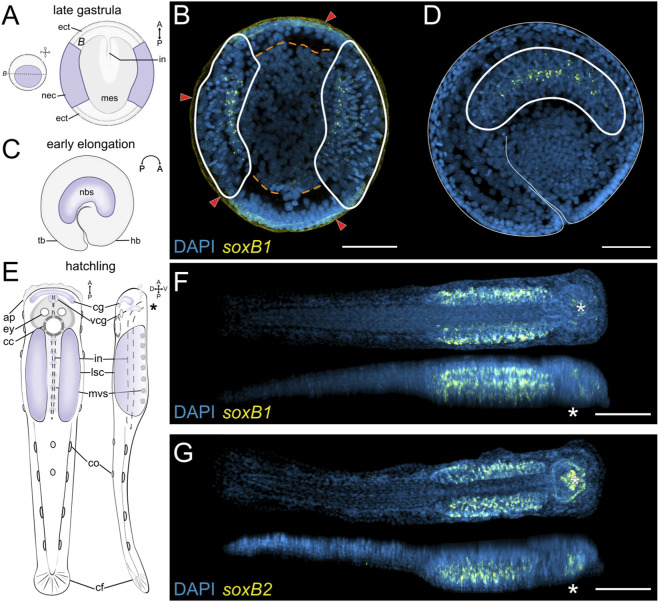
Expression patterns of *Sce-soxB1* and *Sce-soxB2* during embryonic and early post-embryonic development of *Spadella cephaloptera*. **(A,C,E)** Schematic representations of late gastrula **(A)**, early elongation **(C)**, and hatchling **(E)** stages. The late gastrula is shown in dorsal view, the early elongation stage in lateral view, and the hatchling in dorsal (left) and lateral (right) views. Neurogenic regions, including the NEC and neural cells of the VNC, are indicated in purple. **(B,D,F)** *Sce-soxB1* expression. **(B)** Dorsal maximum projection of a late gastrula embryo. Red arrowheads indicate non-specific signal from the inner shell layer. **(D)** Lateral maximum projection of an early elongation embryo. **(F)** Maximum projections of a hatchling, shown in dorsal (top) and lateral (bottom) views. **(G)** *Sce-soxB2* expression in the hatchling. Maximum projections shown in dorsal (top) and lateral (bottom) views. Graphic annotations: White outlines indicate neural territories; orange outlines indicate the endomesoderm in the gastrula stage. Scale bars: 50 μm, except **(F,G)**: 100 µm. Orientation is indicated in the schematic representations. The asterisk marks the position of the mouth. Abbreviations: ap, cephalic adhesive papillae; cc, corona ciliata; cf, caudal fin; cg, cerebral ganglion; co, ciliary tuft/fence organ; ect, ectoderm; epi, epidermis; ey, eye; hb, head bud; in, intestine; lsc, lateral somata clusters; mes, mesodermal cells; mvs, medioventral somata clusters; nbs, neural cells of the developing VNC; nec, neuroectoderm; tb, tail bud; vcg, anteroventral cephalic ganglion anlage.

To determine when a canonical SoxB1 homolog, a marker widely associated with early neuroectodermal and neural progenitor contexts across bilaterians (Buescher et al., 2002; Bylund et al., 2003; Hartenstein and Stollewerk, 2015; Kerner et al., 2009), is first deployed during chaetognath neural development, we examined *Sce-soxB1* expression in *S. cephaloptera*. At the early gastrula stage, *Sce-soxB1* was not detected (Supplementary Figure S10A,B), and expression was not assessed at mid gastrula*.* By the late gastrula stage, *Sce-soxB1* expression appears as bilateral longitudinal strips, predominantly in the inner SNE-cell band (Figures 2A,B). During early elongation, *Sce-soxB1* is expressed in a subset of cells in the nascent lateral somata clusters (Figures 2C,D), and later in the presumptive anteroventral cephalic ganglion anlage, from which the vestibular and esophageal ganglia are inferred to arise. In hatchlings, *Sce-soxB1* expression is present in the eyes, a subset of cells surrounding the base of the future mouth, and the lateral somata clusters (Figures 2E,F; Supplementary Figure S11A-G). Weaker *Sce-soxB1* signal is also observed in the cerebral ganglia and medioventral somata clusters (Figure 2F; Supplementary Figure S11D,F).

#### Sce-soxB2

SoxB2 homologs are commonly associated with later neural developmental contexts in many bilaterians (Guth and Wegner, 2008; Overton et al., 2002). To determine whether a SoxB2-class marker contributes to earlier or later phases of chaetognath neural development, we examined *Sce-soxB2* expression in *S. cephaloptera*. *Sce-soxB2* was not assayed at gastrula stages and was not detected in the early elongation stage (Supplementary Figure S10C). At hatching, *Sce-soxB2* is expressed in the presumptive anteroventral cephalic ganglion anlage, in cells surrounding the future mouth opening, in the presumptive perioral epidermis, and in the lateral somata clusters (Figure 2G; Supplementary Figure S11H-K).

#### *Sce-soxB1-like1* and *Sce-neuroD*

Because *Sce-soxB1* is not detectable until late gastrulation and *Sce-soxB2* is first detected only at hatching, neither gene was suitable for examining SoxB-class expression during the earliest phases of neuroectoderm organization. We therefore used *Sce-soxB1-like1*, the earliest detectable SoxB-related transcript in our dataset, as the primary SoxB marker for characterizing early neurogenic territories and compared it with *Sce-neuroD*, whose orthologs are commonly associated with neural commitment and early neural differentiation in other bilaterians (Bertrand et al., 2002; Deryckere et al., 2021; Sur et al., 2017; 2020), using double HCR labeling.

**FIGURE 3:**
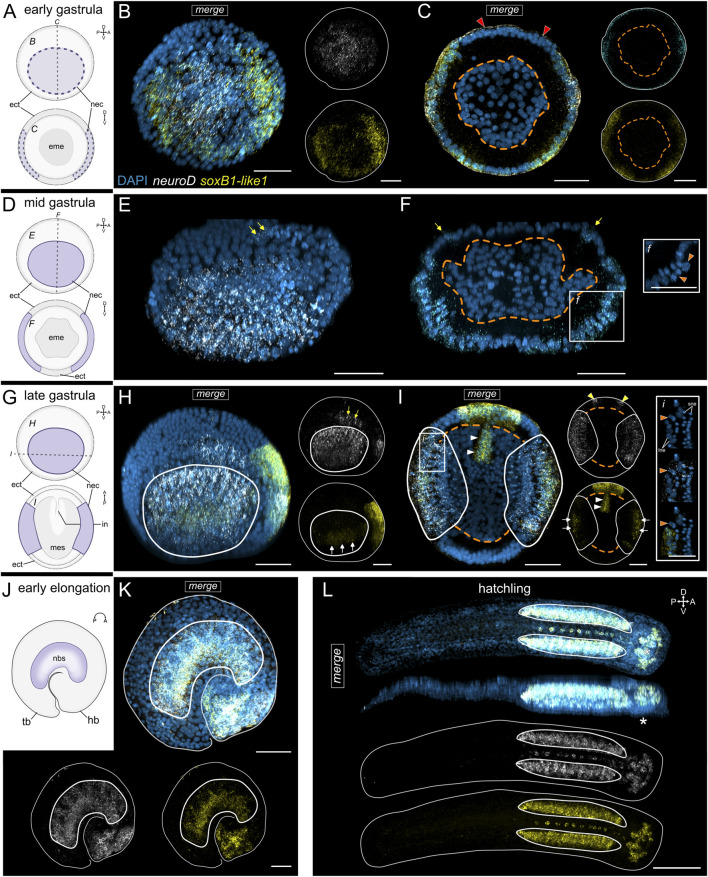
Double-label expression patterns of *Sce-soxB1-like1* and *Sce-neuroD* during embryonic and early post-embryonic development of *Spadella cephaloptera*. Where applicable, fluorescence panels are shown as a merged image (DAPI + probe signals) together with the corresponding individual probe channels. **(A,D,G,J)** Schematic representations of early gastrula **(A)**, mid gastrula **(D)**, late gastrula **(G)**, and early elongation **(J)** stages. Early and mid gastrula stages are shown in lateral (top) and transverse (bottom) views; the late gastrula is shown in lateral (top) and dorsal (bottom) views; the early elongation stage is shown in lateral view. Neurogenic regions, including the NEC and neural cells of the nascent VNC, are indicated in purple. **(B, C)** Early gastrula. **(B)** Lateral maximum projection. **(C)** Transverse section. Red arrowheads indicate non-specific signal from the inner shell layer. **(E,F)** Mid gastrula. **(E)** Lateral maximum projection. **(F)** Transverse section. Yellow arrows indicate expression outside the anatomically defined NEC. Inset *f* shows the DAPI channel from the same field in the merged panel **(F)**. **(H,I)** Late gastrula. **(H)** Lateral maximum projection. Yellow arrows indicate expression detected outside the anatomically defined NEC and white arrows mark the *Sce-soxB1-like1* expression in the ventral NEC. **(I)** Dorsal section. Yellow arrowheads highlight paired anterior ectodermal *Sce-neuroD* expression domains and white arrowheads mark the anterior endomesoderm domain of *Sce-soxB1-like1.* Inset *i* shows corresponding views of DAPI-only staining (top), *Sce-neuroD* (middle), and *Sce-soxB1-like1* (bottom). Orange arrowheads indicate the same nucleus across all channels, which exhibit identifiable mitotic morphology based on the DAPI signal. **(K)** Lateral maximum projection of an early elongation stage embryo. **(L)** Hatchling. Top two rows: merged dorsal and lateral views. Bottom rows: single-channel views. For anatomical context, refer to Figure 2E. The asterisk marks the position of the mouth. Graphic annotations: White outlines indicate neural territories; orange outlines indicate the endomesoderm in gastrula stages; orange arrowheads indicate morphologically identifiable mitotic figures. Scale bars: 50 μm, except **(L)** 100 µm. Orientation is indicated in the schematic representations and, where applicable, in the upper right corner of each panel. Abbreviations: ect, ectoderm; eme, endomesoderm; epi, epidermis; hb, head bud; in, intestine; mes, mesodermal cells; nbs, neural cells of the developing VNC; nec, neuroectoderm; tb, tail bud.

In the early gastrula, *Sce-soxB1-like1* forms a broad lateral domain within the presumptive neuroectodermal region (Figures 3A–C; Supplementary Figure S12D). Within this broader domain, *Sce-neuroD* is expressed in small bilateral mid-lateral patches, such that the *Sce-neuroD* signal lies within the central part of the *Sce-soxB1-like1* territory (Figures 3A–C; Supplementary Figure S12D).

At mid gastrula, *Sce-soxB1-like1* was not assayed. At this stage, *Sce-neuroD* is expressed broadly across the morphologically recognizable presumptive NEC and in a mid-dorsal ectodermal patch that overlies it (Figures 3D–F). Based on its extent relative to the nuclear architecture of the NEC, the *Sce-neuroD* domain appears to include both the LNE and the adjacent SNE populations.

By late gastrula, *Sce-soxB1-like1* is detected within the NEC but is largely confined to the ventral LNE cells (Figures 3H,I; Supplementary Figure S12F). At the same stage, *Sce-neuroD* remains active across the NEC (Figures 3G–I) and in the overlying ectodermal patch (Figure 3H; Supplementary Figure S12E,F). Within the NEC, the *Sce-neuroD* domain appears to extend across the LNE and SNE layers, whereas *Sce-soxB1-like1* overlaps only with the ventral part of this broader domain in the LNE region. In the mid-anterior ectoderm, *Sce-soxB1-like1* forms a plate-like domain that overlaps the anterior bilateral *Sce-neuroD* patches (Figures 3H,I; Supplementary Figure S12E). Additionally, a median anterior endomesodermal domain, presumably within endodermal cells, is positive for *Sce-soxB1-like1* (white arrowheads in Figure 3I; Supplementary Figure S12F).

During early elongation, *Sce-soxB1-like1* and *Sce-neuroD* show extensive overlap in the nascent lateral somata clusters (Figures 3J,K; Supplementary Figure S12G,H) and in the head bud, including in the territory associated with the prospective cerebral ganglia (Supplementary Figure S12H). The dorsal epidermis of the head bud also shows intense *Sce-neuroD* expression (Figure 3K; Supplementary Figure S12H). In the hatchling, *Sce-soxB1-like1* is expressed in the cerebral ganglion, presumptive anteroventral cephalic ganglion anlage, lateral somata clusters, and medioventral somata clusters (Figure 3L; Supplementary Figure S13). *Sce-neuroD* is also expressed in all these neural domains and additionally in the eyes and the corona ciliata (Supplementary Figure S13E).

#### Expression of *bmp2/4* and *chordin*

**FIGURE 4:**
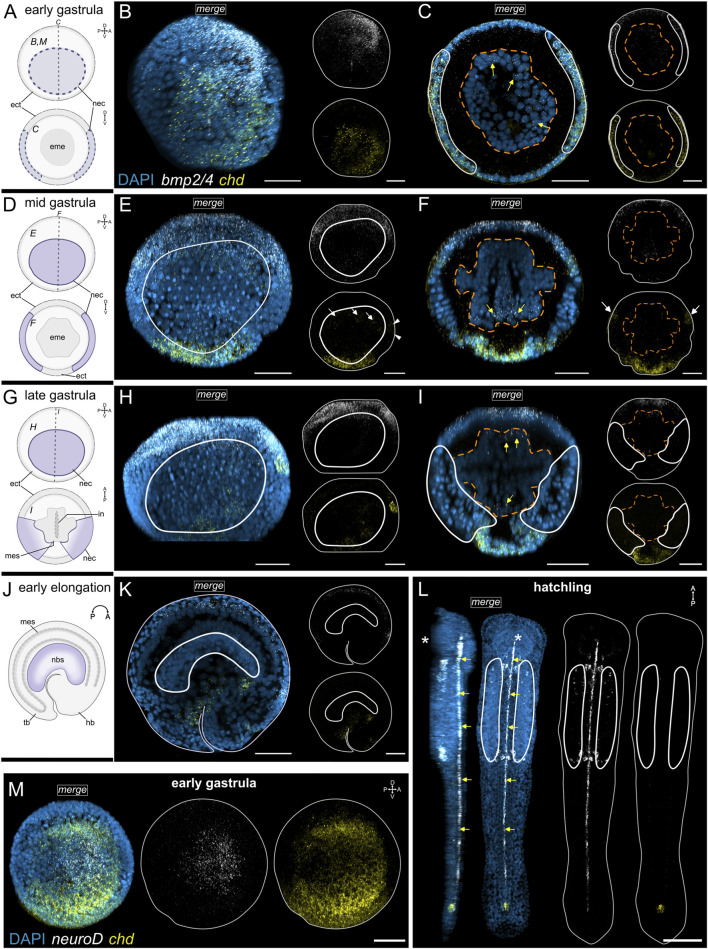
Double-label expression patterns of *Sce-bmp2/4* and *Sce-chd* during embryonic and early post-embryonic development of *Spadella cephaloptera*. Fluorescence panels are shown as merged images (DAPI and two probe signals) together with the corresponding individual probe channels. **(A,D,G,J)** Schematic representations of early gastrula **(A)**, mid gastrula **(D)**, late gastrula **(G)**, and early elongation **(J)** stages. Gastrula stages are shown in lateral (top) and transverse (bottom) views; the early elongation stage is shown in lateral view. Neurogenic regions, including the NEC and neural cells of the nascent VNC, are indicated in purple. **(B,C)** Early gastrula. **(B)** Lateral maximum projection. **(C)** Transverse section. Yellow arrows (in C, F, and I) indicate *Sce-bmp2/4* expression detected in a subset of endomesodermal cells. **(E,F)** Mid gastrula. **(E)** Lateral maximum projection. White arrowheads indicate *Sce-chd* expression in the anterior ectoderm. **(F)** Transverse section. White arrows indicate *Sce-chd* expression within the dorsal portion of the NEC. **(H,I)** Late gastrula. **(H)** Lateral maximum projection. **(I)** Transverse section. **(K)** Lateral section of an early elongation stage embryo. **(L)** Hatchling. Merged lateral (left) and dorsal (right) views and individual probe channels shown in dorsal views. Yellow arrows mark *Sce-bmp2/4* expression in dorsal medial cells. For anatomical context, refer to Figure 2E. **(M)** Maximum projection of double-label expression of *Sce-neuroD* and *Sce-chd* in an early gastrula embryo shown in lateral view. The asterisk marks the position of the mouth. Graphic annotations: White outlines indicate neural territories; orange outlines indicate the endomesoderm in gastrula stages. Scale bars: 50 μm, except **(L)** 100 µm. Orientation is indicated in the schematic representations and, where applicable, in the upper right corner of each panel. Abbreviations: ect, ectoderm; eme, endomesoderm; epi, epidermis; hb, head bud; in, intestine; mes, mesodermal cells; nbs, neural cells of the developing VNC; nec, neuroectoderm; tb, tail bud.

To assess the dorsoventral signaling environment associated with the earliest stages of neuroectoderm organization, we analyzed the expression of *Sce-bmp2/4* and *Sce-chd*. In the early gastrula, *Sce-bmp2/4* is expressed in the dorsal ectoderm (Figures 4A–C; Supplementary Figure S14A-C) and in a subset of endomesodermal cells (yellow arrows in Figure 4C). In contrast, *Sce-chd* expression occupies a broad domain spanning the lateral ectoderm and extending across the ventral region (Figures 4A–C; Supplementary Figure S14A-C). Because *Sce-chd* shows its broadest ectodermal extent in the early gastrula, and this is also the earliest stage at which a discrete *Sce-neuroD* domain can be identified, we examined *Sce-chd* and *Sce-neuroD* together by double HCR at this stage to assess their spatial relationship during the initial emergence of the neuroectoderm. Double HCR shows that *Sce-neuroD* expression lies within the *Sce-chd+* lateral ectoderm, including the presumptive neuroectodermal region (Figure 4M; Supplementary Figure S12B,C).

*Sce-bmp2/4* expression continues to mark the dorsal ectoderm and extends posteriorly towards the blastoporal region in the mid gastrula (Figures 4D–F; Supplementary Figure S14D-G), with persistent expression in some cells of the endomesoderm (yellow arrows in Figure 4F; Supplementary Figure S14G). During this stage, *Sce-chd* expression shows reduced ectodermal signal and is mainly detected in the ventral ectoderm (Figures 4D–F; Supplementary Figure S14F), with faint expression persisting in the NEC (Figure 4E, F; Supplementary Figure S14G), and a low-level signal in the mid-anterior ectoderm (Figure 4E; Supplementary Figure S14G).

By the late gastrula stage, *Sce-bmp2/4* expression persists in the dorsal ectoderm, extending from the mid-anterior region toward the posterior ectoderm (Figures 4G–I; Supplementary Figure S14H-K). *Sce-bmp2/4* expression also remains detectable in a subset of presumably mesodermal cells (yellow arrows in Figure 4I). *Sce-chd* expression further weakens in overall intensity and is largely restricted to the ventral ectoderm, with an additional bilateral pair of patches appearing in the mid-anterior ectoderm (Figures 4G–I; Supplementary Figure S14J,K).

In early elongation, *Sce-bmp2/4* is expressed in the dorsal ectoderm of the head bud, in mesodermal cells along the dorsal region of the developing trunk (Figures 4J,K). *Sce-chd* shows patchy expression in the ventral and dorsal head bud, the ventral trunk, and tail bud. At hatching, *Sce-bmp2/4* is expressed in the dorsal medial cells forming a continuous longitudinal line that extends from the base of the head to the posterior tail, in ventral cells of the anterior and posterior boundaries of the trunk, and in a few cells of the lateral somata clusters (Figure 4L; Supplementary Figure S14L-Q). In contrast, *Sce-chd* expression is restricted to a small patch in the posterior tail (Figure 4L).

#### Expression of *hb9* and *nk6*

**FIGURE 5:**
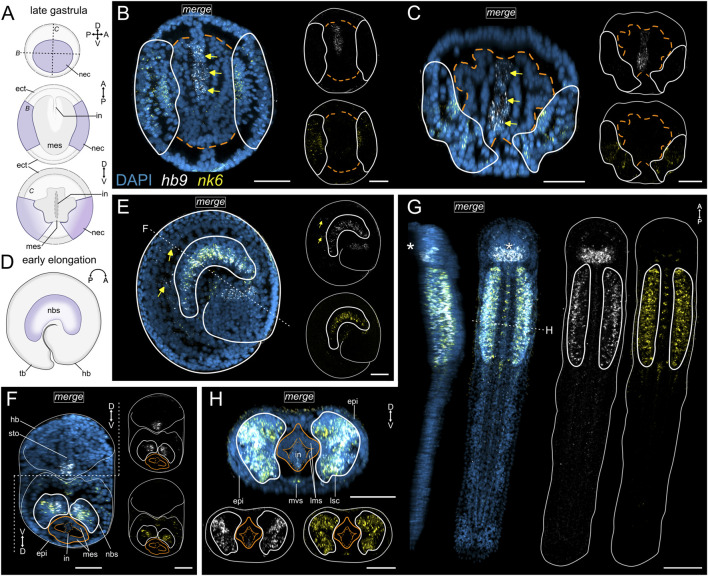
Double-label expression patterns of *Sce-hb9* and *Sce-nk6* during embryonic and early post-embryonic development of *Spadella cephaloptera*. Fluorescence panels are shown as merged images (DAPI and two probe signals) together with the corresponding individual probe channels. **(A,D)** Schematic representations of late gastrula **(A)** and early elongation **(D)** stages. The late gastrula is shown in lateral (top), dorsal (middle) and transverse (bottom) views; the early elongation stage is shown in lateral view. Neurogenic regions, including the NEC and neural cells of the nascent VNC, are indicated in purple. **(B,C)** Late gastrula. Dorsal **(B)** and transverse sections **(C). (E,F)** Early elongation. **(E)** Lateral maximum projection. White arrows indicate *Sce-hb9* expression in the trunk region. **(F)** Transverse section through the early elongation trunk highlighting the dorsoventral expression domains of *Sce-hb9* and *Sce-nk6* within the nascent VNC. **(G,H)** Hatchling. **(G)** Maximum projection shown in lateral (left) and dorsal (right) views. For anatomical context, refer to Figure 2E. **(H)** Transverse section through the hatchling trunk highlighting the expression domains of *Sce-hb9* and *Sce-nk6* within the lateral somata clusters. The asterisk marks the position of the mouth. Graphic annotations: White outlines indicate neural territories; orange outlines indicate the endomesoderm in gastrula stages and mesodermal cells and the intestinal region in the early elongation stage and hatchling. Scale bars: 50 μm, except **(G)** 100 µm. Orientation is indicated in the schematic representations and, where applicable, in the upper right corner of each panel. Abbreviations: ect, ectoderm; eme, endomesoderm; epi, epidermis; hb, head bud; in, intestine; lms, longitudinal muscle somata; mes, mesodermal cells; mvs, medioventral somata clusters; nbs, neural cells of the developing VNC; nec, neuroectoderm; sto, stomodeum; tb, tail bud.

Across bilaterians, *nk6* and *hb9* orthologs are widely associated with ventral neural patterning (Arber et al., 1999; Cheesman et al., 2004; Denes et al., 2007; Schmidt et al., 1997). We therefore analyzed *Sce-nk6* and *Sce-hb9* to assess the early regionalization of ventral neuronal subtype-associated domains in *S. cephaloptera*. Expression of *Sce-hb9* and *Sce-nk6* was not examined at earlier stages (early and mid gastrula) in the present study. At the late gastrula stage, *Sce-hb9* is expressed in the presumptive endodermal cells, whereas *Sce-nk6* expression is broadly detected in the midventral SNE-cell territory (Figures 5A–C). During early elongation, *Sce-hb9* is expressed in the nascent lateral somata clusters and in the ventral stomodeum (Figures 5D–F). Expression is also observed in scattered cells of the developing trunk (Figure 5E). *Sce-nk6* is similarly expressed in the nascent lateral somata clusters, occupying a more dorsal domain that partially overlaps with *Sce-hb9* expression (Figures 5E,F). At hatching, *Sce-hb9* is expressed in the presumptive anteroventral cephalic ganglion anlage, perioral epidermis, lateral somata clusters and the gut (Figures 5G,H; Supplementary Figure S15A-D,F), while *Sce-nk6* expression is detected in some cells of the head, medioventral somata clusters, the trunk epidermis, and in cells near the trunk–tail boundary (Figures 5G,H; Supplementary Figure S15C-H). Within the lateral somata clusters, the *Sce-hb9* domain occupies most of the ventral and mid regions, whereas *Sce-nk6* expression spans a broader domain that includes this the dorsal territory lacking detectable Sce-hb9^+^ expression (Figure 5H).

#### Expression of *th* and *dbh*

**FIGURE 6:**
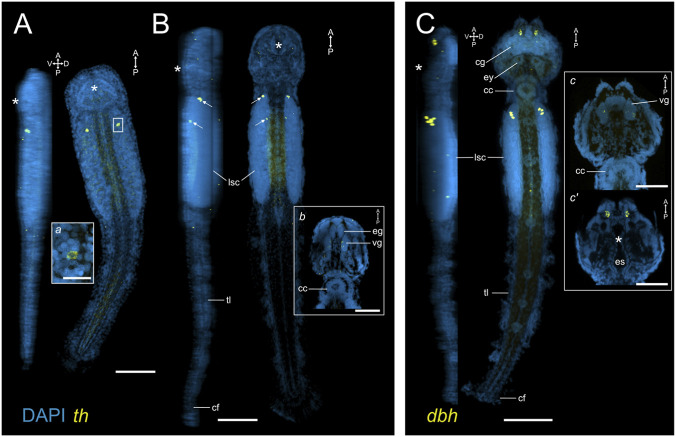
Expression patterns of *Sce-th* and *Sce-dbh* during early post-embryonic development of *Spadella cephaloptera.* **(A,B)** *Sce-th* expression. **(A)** Maximum projection of a hatchling in lateral (left) and dorsal view (right). Inset *a* shows a magnified view of one of the two *Sce-th* ^*+*^ cells. For anatomical context, refer to Figure 2E. **(B)** Maximum-intensity projection of an early juvenile in lateral (left) and dorsal (right) view. White arrows indicate *Sce-th* ^+^ cells within the lateral somata clusters. Inset *b* shows a dorsal section of the head highlighting *Sce-th* expression in the vestibular ganglia. The asterisk marks the position of the mouth. **(C)** *Sce-dbh* expression. Maximum projection of an early juvenile in lateral (left) and dorsal view (right). Insets *c* and *c′* show dorsal sections of the head at different dorsoventral levels. Inset *c* corresponds to a more dorsal section highlighting *Sce-dbh* expression in the vestibular ganglia, whereas inset *c′* shows a more ventral level encompassing the anterior mouth region. Scale bars: 100 μm, except **(A)** 25 μm; (insets b, c, c′): 50 µm. Orientation is indicated in the upper right corner of each panel. Abbreviations: cc, corona ciliata; cf, caudal fin; cg, cerebral ganglion; eg esophageal ganglia; ey, eye; es, esophagus; lsc, lateral somata clusters; tl, tail; vg, vestibular ganglia.

To assess the onset of catecholaminergic differentiation during chaetognath neural development, we characterized the expression of *Sce-th* and *Sce-dbh*, which encode key enzymes in catecholamine biosynthesis (Goulty et al., 2023; Sloley and Juorio, 1995). Embryonic stages were not examined for *Sce-th* and *Sce-dbh* expression in the present study. At hatching, *Sce-th* is expressed in a single bilateral pair of neurons in the anterior lateral somata clusters, with a second pair appearing in early juveniles (Figures 6A,B). *Sce-th* expression is also detected in the vestibular ganglia in the early juvenile (inset in Figure 6B). *Sce-dbh* expression is not detected in hatchlings (Supplementary Figure S10D). In early juveniles, *Sce-dbh* expression appears in three bilateral pairs of cells within the anterior lateral somata clusters and in cells of the anterior mouth (Figure 6C). The vestibular ganglia also show low-level *Sce-dbh* expression (inset in Figure 6C).

### Negative controls for HCR assays

Hairpin-only negative controls showed no specific signal at the stages examined under the assay and imaging conditions used in this study (Supplementary Figure S16).

## Discussion

This study examines the spatial and temporal domains of broadly conserved developmental genes associated with nervous system formation in the chaetognath *S. cephaloptera*. Spiralian nervous systems span substantial architectural diversity, and chaetognaths possess a compact nervous system with a comparatively distinctive gross organization (Harzsch and Wanninger, 2010; Rieger et al., 2011; 2010), thereby providing a valuable context for assessing how conserved molecular patterning signatures are spatiotemporally deployed within this lineage.

**FIGURE 7:**
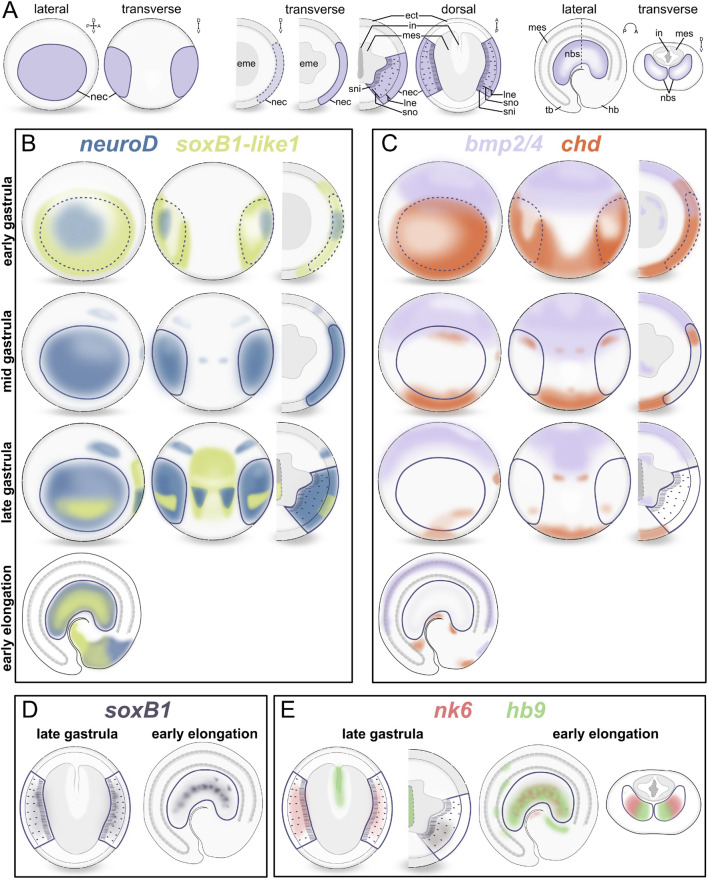
Schematic representation of neural-associated and dorsoventral patterning gene expression during embryonic development of *Spadella cephaloptera*. **(A)** Anatomical reference schematics showing whole gastrula (lateral and dorsal views) and representative sections (transverse sections of early, mid, and late gastrula; dorsal section of late gastrula; and lateral and transverse sections through the trunk at early elongation). Neurogenic regions, including the neuroectoderm (NEC) and neural cells of the developing ventral nerve cord (VNC), are indicated in purple. At the early gastrula stage, the purple domain corresponds to the presumptive neuroectoderm. At the late gastrula stage, the three subdivision of nuclei is shown: LNE cells in the outermost layer, middle SNE layer (sno), and the inner more compact SNE layer (sni). **(B)** Summary of *Sce-soxB1-like1* and *Sce-neuroD* expression domains across gastrula and early elongation stages. **(C)** Summary of *Sce-bmp2/4* and *Sce-chd* expression domains across gastrula and early elongation stages. **(D)** Summary of *Sce-soxB1* expression domains in late gastrula and early elongation. **(E)** Summary of *Sce-nk6* and *Sce-hb9* expression domains in late gastrula and early elongation. Abbreviations: ect, ectoderm; eme, endomesoderm; hb, head bud; in, intestine; lne, large-nucleus neuroectodermal cell layer mesoderm; nbs, neural cells of the developing VNC; nec, neuroectoderm; sni, inner layer of small-nucleus cells; sno, outer layer of small-nucleus cells; tb, tail bud.

Our analyses identify molecularly distinguishable domains corresponding to early neurogenic territories, dorsoventral patterning context, and the initial differentiation of neuronal subtypes (Figure 7). These expression-based data map the emergence of discrete neural territories and neuronal subtypes in *S. cephaloptera*, highlighting both commonly observed bilaterian patterns and lineage-specific features of nervous system development.

### Early neuroectodermal organization and neurogenic progression in *Spadella*

Classical embryological descriptions of *S. cephaloptera* noted how the cells of the VNC originate from the ventrolateral ectoderm of the embryo, but did not resolve the identity of the neuroectodermal precursors that give rise to these neurons, nor clarify how early neurogenic cell populations are established (John, 1933). Here, we integrate *Sce-soxB1-like1* and *Sce-neuroD* expression with DAPI-based nuclear morphology to define an early neuroectodermal territory during gastrulation and to compare its molecular organization across successive developmental stages (Figure 3; Figure 7B).

The broad domain of *Sce-soxB1-like1* expression in the early gastrula spatially coincides with an ectodermal region characterized by comparatively large, relatively DAPI-faint nuclei and frequent mitotic figures. Although reduced DAPI intensity and large nuclei have been associated in some systems with less condensed chromatin states and proliferative or progenitor-like populations (Faro-Trindade and Cook, 2006; Madl et al., 2020), these features alone are not sufficient to establish progenitor identity. We therefore interpret this *Sce-soxB1-like1*+ territory as a putative early neurogenic or neural-competent domain, based primarily on its spatial extent and anatomical context. This interpretation is consistent with the widespread association of SoxB1 orthologs with early neuroectodermal territories across bilaterians (e.g., Sox1/2/3 in vertebrates and SoxNeuro in arthropods), including in diverse spiralian lineages (Buescher et al., 2002; Bylund et al., 2003; Deryckere et al., 2021; Hartenstein and Stollewerk, 2015; Kerner et al., 2009; Kurtova et al., 2024; Monjo and Romero, 2015b).

Notably, weak *Sce-neuroD* signal is already detectable within the *Sce-soxB1-like1+* territory, reinforcing the view that this region represents an early neurogenic domain. By mid-gastrulation, *Sce-neuroD* is expressed broadly across the morphologically recognizable presumptive NEC. At this stage, SNE cells emerge adjacent to and interspersed among the LNE cells, and thus the Sce-neuroD domain is present in both cell populations of the NEC, including in cells of the LNE-cell population that show mitotic activity (Figure 3F). Together, these observations support the interpretation that *Sce-neuroD* is deployed broadly at an early stage of NEC organization. Because *Sce-soxB1-like1* expression was not characterized at mid-gastrula, this limits our ability to assign temporal relationships between broad *SoxB1-like1* expression and the onset of broad *Sce-neuroD* expression at this stage.

By late gastrulation, the two markers show a more resolved spatial relationship, with *Sce-neuroD* expression maintained across the LNE and SNE layers and *Sce-soxB1-like1* restricted to the ventral portion of the NEC, where their expression domains overlap within the LNE population (Figures 3G–I; Figure 7B). Frequent mitotic figures are also present within the LNE cell population at this stage, indicating that broad *Sce-neuroD* expression persists in cells that still exhibit overt mitotic activity. Early deployment of NeuroD-family genes in proliferative neuroectodermal or progenitor populations has been reported in several spiralian taxa, including the annelid *Platynereis dumerilii* and planarians (Cowles et al., 2013; Monjo and Romero, 2015a; Simionato et al., 2008). By contrast, in other bilaterians such as the annelid *Capitella teleta*, the cephalopod *Octopus vulgaris*, and vertebrates, NeuroD-family genes are predominantly associated with terminally dividing or post-mitotic neural precursors (Bertrand et al., 2002; Deryckere et al., 2021; Sur et al., 2017; 2020). Therefore, the *Sce-neuroD* expression profile suggests that the timing of *neuroD* deployment during chaetognath neurogenesis may differ from systems in which NeuroD-family expression is restricted to later neural precursors, although direct assessment of its relationship to cell proliferation will require co-labeling with proliferation markers, such as phospho-Histone H3 or EdU.

SNE cells also increase in number and become positioned more internally during late gastrulation, accumulating near the ventrolateral borders of the endomesoderm (Figure 1H). This transition coincides with the emergence of detectable *Sce-soxB1* expression within a subset of the inner SNE population. This expression is arranged in a repeated pattern along the anterior–posterior axis rather than uniformly across the neuroectoderm (Figure 2; Figure 7D), and a similarly restricted pattern is maintained during early elongation in subsets of cells within the developing lateral somata clusters. Although *Sce-soxB1-like1* expression is also spatially restricted at late gastrula, being largely confined to ventral LNE cells, the two SoxB1 paralogs occupy distinct subdomains at this stage. By early elongation, this difference becomes more pronounced, as *Sce-soxB1-like1* expression broadens across the nascent VNC and shows extensive overlap with the *Sce-neuroD* expression domain, whereas *Sce-soxB1* remains restricted to a subset of cells. Together, these data suggest that the molecular regionalization during gastrulation remains evident during formation of the VNC, while the spatial relationship between the two SoxB1 paralogs changes as the chaetognath CNS becomes morphologically more defined. Similar spatiotemporal partitioning among SoxB1 paralogs has been reported in other bilaterians, including several vertebrate representatives and the planarian *Schmidtea polychroa*, suggesting that paralog-specific divergence in timing and regional expression is a recurring feature of neural development (Meulemans and Bronner-Fraser, 2007; Monjo and Romero, 2015a; Okuda et al., 2006).

### Molecular characterization of a putative anterior neuroectodermal territory

In addition to the ventrolateral neuroectoderm associated with VNC formation, we identify a discrete molecular territory marked by *Sce-neuroD* and *Sce-soxB1-like1* during late gastrulation and early elongation. In the late gastrula, *Sce-neuroD* expression forms paired anterior domains that overlap with the broader anterior ectodermal expression of *Sce-soxB1-like1* (Figures 3G–I; Figure 7A). This territory lies within the anterior ectodermal field marked by *Sce-otx*, providing additional positional support for a potential anterior neuroectodermal identity (Ordoñez and Wollesen, 2025). During early elongation, a bilateral *Sce-neuroD* ^*+*^/*Sce-soxB1-like1* ^+^ territory is observed on both sides of the anterior head bud (Supplementary Figure S12H). This position closely matches the thickened anterior neuroectoderm in the early elongation stage described in classical embryological studies as contributing to the cerebral ganglia (John, 1933). However, definitive assignment of this territory to the presumptive head neuroectoderm will require experimental validation, such as lineage tracing.

### Overlapping proliferative and differentiation-associated transcriptional signatures in the hatchling nervous system

Ultrastructural and immunohistochemical context reveals that *S. cephaloptera* hatchlings emerge with a nervous system that is functionally competent yet developmentally immature. Rieger et al. (2011) described the VNC containing differentiated neurons, including RFamide+ and synapsin+ populations, while noting that synaptic networks, commissural tracts, and vesicle complements remain sparse and continue to expand post-hatching. Our molecular data are consistent with this interpretation, as the same neural regions express transcriptional markers spanning multiple neurogenic states, including early neurogenic programs (*Sce-neuroD*, *Sce-soxB2*) and markers associated with neuronal maturation (*Sce-hb9*, *Sce-th*, *Sce-dbh*). Broad *Sce-elav* expression in the hatchling VNC and brain (Ordoñez and Wollesen, 2024) further supports the presence of neurons that have entered molecular stages of differentiation.

In addition, the hatchling brain and VNC remain mitotically active for several days post-hatching, with BrdU+ nuclei forming bilateral bands along the inner margins of the lateral somata clusters (Perez et al., 2013). When considered alongside our molecular data, these proliferative cells correspond to neural populations expressing gene complements typically associated with early neurogenic or fate-biased precursor states rather than naïve neuroectoderm. This configuration contrasts with many metazoan systems, including cnidarians, insects, chordates, and *Capitella*, in which NeuroD- and Elav-family genes are largely restricted to post-mitotic differentiating neurons (D’Amico et al., 2013; Kim et al., 1996; Nakanishi et al., 2012; Robinow and White, 1991; Sur et al., 2017). The coexistence of proliferative activity and differentiation-associated markers is therefore consistent with a population of transit-amplifying or lineage-restricted neural precursors interspersed with differentiating neurons, rather than a large undifferentiated progenitor pool. Whether these proliferative cells derive from persisting embryonic neuroectoderm or represent a distinct progenitor population remains unresolved. Overall, the hatchling VNC integrates proliferative, differentiating, and mature neuronal features within a compact neural architecture that continues to develop after hatching.

In this context, the differential deployment of three SoxB paralogs provides molecular insight into the neurogenic landscape of the hatchling CNS. *Sce-soxB1-like1* is broadly expressed across hatchling neural structures, consistent with activity in differentiating neural territories. This pattern aligns with observations from multiple bilaterian systems in which SoxB1-type genes function not only in neural competence but also during later stages of neural differentiation (Buescher et al., 2002; Deryckere et al., 2021; Ekonomou et al., 2005; Ferrero et al., 2014; Monjo and Romero, 2015a; Overton et al., 2002; Sur et al., 2017; Vidal et al., 2015). By contrast, *Sce-soxB1* shows a more spatially restricted expression pattern, being localized to the presumptive anteroventral cephalic ganglion anlage and to discrete subpopulations of the lateral somata clusters, with weaker expression in parts of the cerebral ganglion and medioventral somata clusters. This distribution suggests that *Sce-soxB1* is enriched in specific neural subdomains rather than broadly marking differentiated neurons, potentially reflecting roles in neural lineage refinement or regional specification. Unlike the two SoxB1 paralogs, *Sce-soxB2* expression is first detected at the hatchling stage, specifically within the lateral somata clusters and the developing ventral cephalic ganglia. This late onset is consistent with the deployment of SoxB2 homologs in several bilaterian lineages, where these transcription factors are more frequently associated with neural differentiation and subtype specification rather than progenitor maintenance (Guth and Wegner, 2008; Monjo and Romero, 2015a; Overton et al., 2002; Wegner and Stolt, 2005; Zhao and Skeath, 2002). Thus, the distinct expression profiles of *Sce-soxB1-like1*, *Sce-soxB1*, and *Sce-soxB2* across neural territories exhibiting features of both proliferative activity and maturation provide pattern-level support for the idea that SoxB family members can act at multiple stages of neurogenesis, as proposed in other bilaterian systems.

### Bmp2/4-chordin dorsoventral patterning during early neuroectodermal specification

In many bilaterians, BMP signaling, modulated by antagonists of the Chordin family and related BMP inhibitors, constitutes a core dorsoventral (DV) patterning system and is often associated with the positioning of neuroectodermal territories relative to the surrounding ectoderm (Bier and De Robertis, 2015; Erwin, 2009). In *S. cephaloptera, Sce-bmp2/4* and *Sce-chordin* exhibit a reciprocal DV expression pattern in the early gastrula, coinciding with the stage at which the neuroectodermal markers, *Sce-soxB1-like1* and *Sce-neuroD,* are first detected in our dataset (Figure 4; Figure 7C). These neural markers arise within the lateral to ventrolateral regions of the embryo that correspond to *Sce-chordin* ^+^ and *Sce-bmp2/4*-reduced domains. The BMP pathway activity at this stage is reflected by dorsally localized phosphorylated Smad1/5/8 (pSmad1/5/8), indicating active BMP signaling output (Knabl et al., 2025). Notably, pSmad1/5/8 does not fully coincide with *Sce-bmp2/4* transcript domains, a pattern that is expected in BMP–Chordin systems, where extracellular modulation, including ligand diffusion and antagonistic interactions, shapes signaling fields that extend beyond sites of ligand (i.e., *Sce-bmp2/4*) transcription (Rahman et al., 2015; Zakin and De Robertis, 2010). These observations indicate the presence of a dorsoventrally polarized BMP signaling environment during gastrulation, at the time early neurogenic territories become molecularly distinguishable.

This arrangement parallels patterns in several bilaterian systems, including lophotrochozoans, insects, and vertebrates, in which regions of relatively low BMP activity or elevated *chordin* expression are frequently associated with territories permissive for neural competence, whereas BMP-high domains typically correspond predominantly to non-neural ectoderm (Denes et al., 2007; Holley et al., 1995; Lewin et al., 2026; Martín-Durán et al., 2018; Su et al., 2019; Tan et al., 2022). At the same time, substantial variability of DV patterning mechanisms has also been reported across spiralian lineages, including cases in which canonical BMP–Chordin antagonism is modified or replaced (Kuo and Weisblat, 2011; Lanza and Seaver, 2020; 2018; Lyons et al., 2020).

As gastrulation proceeds, DV expression domains in *S. cephaloptera* become narrower, with *Sce-chordin* expression progressively retracting ventrally and *Sce-bmp2/4* persisting dorsally and in endomesodermal cells. By early elongation, these genes no longer form broad axial gradients and, by the hatchling stage, both genes are restricted to discrete cell populations. This progression indicates that DV patterning in *S. cephaloptera* is temporally restricted to gastrulation, coinciding with the establishment of initial neurogenic territories, and that *Sce-bmp2/4* and *Sce-chordin* are subsequently redeployed in more localized and tissue-specific roles.

Importantly, BMP signaling is not universally excluded from neural territories and can be active within developing nervous systems of cnidarians and bilaterians, including *S. cephaloptera* (Carrillo-Baltodano et al., 2025; Knabl et al., 2025; Moore et al., 2025; Mörsdorf et al., 2024; Pasini et al., 2006; Rusten et al., 2002; Waki et al., 2015). In several systems, BMP activity has been implicated in aspects of neurogenic regulation, including neuronal differentiation or subtype patterning, rather than acting solely as an anti-neural signal (Denes et al., 2007; Knabl et al., 2025; Lambert et al., 2016; McClay et al., 2018; Tan et al., 2022). Taken together, our data support a model in which *S. cephaloptera* shows an early and transient DV BMP–Chordin expression framework associated with neuroectodermal emergence, followed by a later, more localized deployment of *Sce-chordin*, *Sce-bmp2/4*, and associated BMP pathway components within specific tissues, including neural territories.

### *Nk6* and *hb9* expression patterns indicate early motor neuron-associated ventral patterning

To investigate how ventral neuronal subtypes emerge in *S. cephaloptera*, we characterized the expression patterns of *Sce-nk6* and *Sce-hb9*, two conserved transcription factors widely implicated in ventral CNS patterning and motor neuron specification across bilaterians (Arber et al., 1999; Cheesman et al., 2004; Denes et al., 2007; Dichmann and Harland, 2011; Ferrier et al., 2001; Odden et al., 2002; Schmidt et al., 1997). In *S. cephaloptera*, *Sce-nk6* expression is detectable by late gastrulation in a subset of SNE cells within the midventral neuroectoderm, indicating that molecular subdivision within the neurogenic field is already initiated at this stage (Figure 5; Figure 7D). This ventral bias becomes more apparent during early elongation, when *Sce-nk6* and *Sce-hb9* jointly mark the developing VNC in partially overlapping domains. In hatchlings, *Sce-hb9* expression is detected in the presumptive anteroventral cephalic ganglion anlage, consistent with previous interpretations that the vestibular ganglia accommodate motor neurons innervating the head musculature of chaetognaths (Müller et al., 2018; Rieger et al., 2011; Shinn, 1997). Notably, within the lateral somata clusters, a predominantly ventral *Sce-hb9* domain occupies a subset of a broader Sce*-nk6* *+* territory. This overlapping *Sce-nk6*+/*Sce-hb9*+ organization is consistent with conserved ventral CNS patterning schemes, in which Nk6-family transcription factors mark ventral progenitor or precursor domains that give rise to motor neurons and specific ventral interneuron subtypes, while *hb9*/*mnx1* (*motor neuron and pancreas homeobox 1*) functions as a terminal selector for motor neuron identity (Arber et al., 1999; Cheesman et al., 2004; Denes et al., 2007; Dichmann and Harland, 2011; Ferrier et al., 2001; Odden et al., 2002; Schmidt et al., 1997). Moreover, the spatial deployment of *Sce-nk6* and *Sce-hb9* during development further provides molecular support for a mediolateral organization of the VNC previously proposed for *S. cephaloptera* (Ordoñez and Wollesen, 2024).

Although our analysis focuses on neural territories, *Sce-hb9* is also expressed in the presumptive endoderm at late gastrulation and later in the hatchling gut. Comparable dual expression of *hb9*/*mnx1* expression in both the CNS and the gut has been reported in *Drosophila* (Odden et al., 2002) and across deuterostomes, including sea urchin, amphioxus, and vertebrates (Bellomonte et al., 1998; Harrison et al., 1994; 1999). This shared neural–endodermal expression pattern therefore appears to represent an evolutionarily conserved feature of *hb9/mnx1* deployment across Bilateria (Bellomonte et al., 1998; Ferrier et al., 2001; Harrison et al., 1999; 1994; Saha et al., 1997).

### Post-embryonic deployment of catecholamine pathway components

Tyrosine hydroxylase (TH) and dopamine β-hydroxylase (DBH) encode core enzymes in catecholamine biosynthesis, and their biochemical activities are conserved throughout Bilateria (Goulty et al., 2023; Sloley and Juorio, 1995). Both enzymes have been widely used as molecular markers associated with monoaminergic neuron identity, including dopaminergic and noradrenergic neurons, in well-characterized systems (Flames and Hobert, 2011). In the *S. cephaloptera* hatchling, *Sce-th* expression is detected in a small bilateral subset of VNC cells, whereas *Sce-dbh* is not detected at this stage. *Sce-dbh* expression is first detected in early juveniles, in the anterior VNC and head region. This temporal offset suggests that catecholamine-associated molecular components are activated sequentially during early post-embryonic development. In the early juvenile, *Sce-dbh* is restricted to the anterior-most region of the VNC with additional non-neural expression in the head region. While this region may later contribute to anterior mouth structures and associated sensory elements, the cellular identity and functional significance of these anterior head domains remain unresolved at this stage.

At present, direct comparisons with other spiralians remain limited due to the scarcity of molecular-level data on DBH deployment in this group. Non-neuronal *dbh* expression has only been reported in a small number of spiralian species, including hemocytes and liver tissue in bivalve mollusks (Li et al., 2021; Zhou et al., 2011) and protein-level localization in the urn cells of the dicyemid *Dicyema japonicum* (Lu et al., 2019). Nevertheless, these observations place the *S. cephaloptera* data within a broader but still sparse comparative context and support the conclusion that *Sce-th* and *Sce-dbh* show temporally and spatially distinct expression patterns during early post-embryonic development.

## Conclusion

Our analysis shows that nervous system development in *S. cephaloptera* relies on conserved bilaterian molecular programs that are deployed in lineage-specific spatial and temporal patterns. *Sce-soxB* and *Sce-neuroD* expression delineate a neuroectodermal territory in which proliferative and differentiation-associated programs overlap, while a transient *Sce-bmp2/4* - *Sce-chordin* dorsoventral pattern coincides with the emergence of early neurogenic territories. Ventral neuronal subtype patterning, including early motor-neuron specification marked by *Sce-nk6* and *Sce*-*hb9*, further supports the conservation of core bilaterian neural patterning mechanisms in chaetognaths. In contrast, the restricted expression of *Sce-th* and the delayed onset of *Sce-dbh* indicate that catecholaminergic pathway components are assembled progressively within established neural territories rather than as a unified monoaminergic system. Collectively, these findings define the spatial and temporal organization of early neurogenesis in chaetognaths and provide a comparative reference for future analyses of how markers associated with bilaterian neurogenesis are deployed across spiralian lineages.

## Data Availability

The assembled transcriptome for Spadella cephaloptera is accessible on Zenodo (https://zenodo.org/record/7602960#.Y90U0oSZOUk/DOI:10.5281/zenodo.7602960). Newly obtained *S. cephaloptera* sequences used in the phylogenetic analyses have been deposited to GenBank (accession numbers: PZ054574 - PZ054584). Final alignment files used in orthology analyses are provided in the Supplementary Data.
