# Supplementary material for: Early nervous system development in the chaetognath Spadella cephaloptera exhibits conserved bilaterian patterning features
Source: Front Cell Dev Biol. 2026 May 28;14:1819435. doi: 10.3389/fcell.2026.1819435 (PMC13253686; doi:10.3389/fcell.2026.1819435)
Supplement: Supplementary file 2 [file DataSheet1.docx]

**Supplementary Figures**

**Early nervous system development in the chaetognath *Spadella cephaloptera* exhibits conserved bilaterian patterning features**

June F. Ordoñez^1,2,^*, Alice Frisinghelli^1,2^, Cristian Camilo Barrera Grijalba^1,2^, Tim Wollesen^1,^*

*1 Unit for Integrative Zoology, Department of Evolutionary Biology, University of Vienna, 1030 Vienna, Austria*

*2 Vienna Doctoral School of Ecology and Evolution (VDSEE), University of Vienna, Austria*

*Corresponding authors

Tim Wollesen

Email address: tim.wollesen@univie.ac.at

June F. Ordoñez

Email address: june.ordonez@univie.ac.at


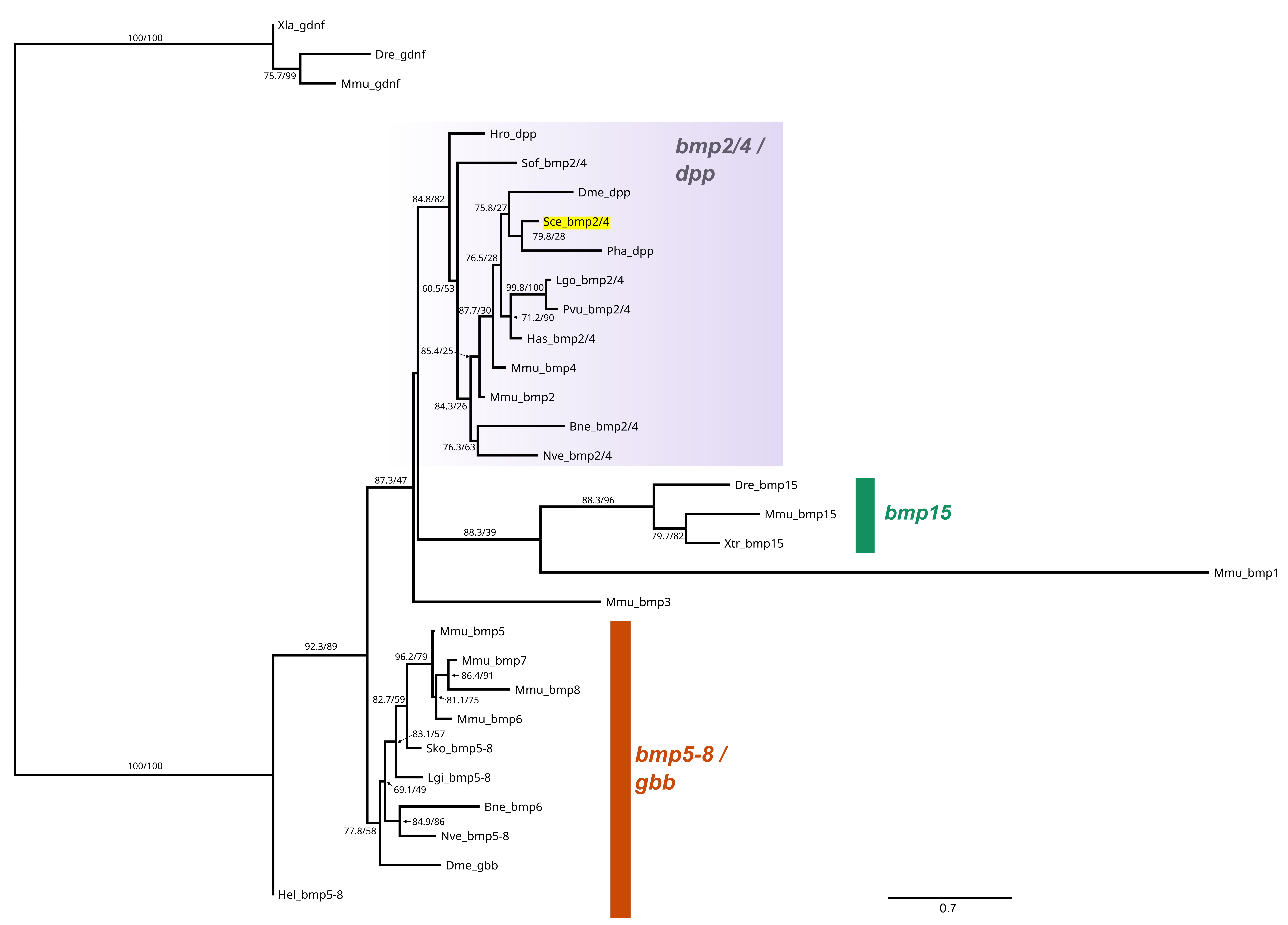


**Supplementary Figure 1.** Maximum Likelihood phylogenetic analysis of Bmp2/4/Dpp and related BMP-family amino acid sequences, including the deduced Bmp2/4/Dpp protein from *Spadella cephaloptera* (highlighted in yellow). The tree was inferred using IQ-TREE based on bilaterian protein sequences obtained from published sources and NCBI GenBank BLAST searches. Branch support values are shown as SH-aLRT (%) / ultrafast bootstrap support (UFBS %). Support labels are omitted for nodes where both values are <50. Species abbreviations are provided in Supplementary Table 1.


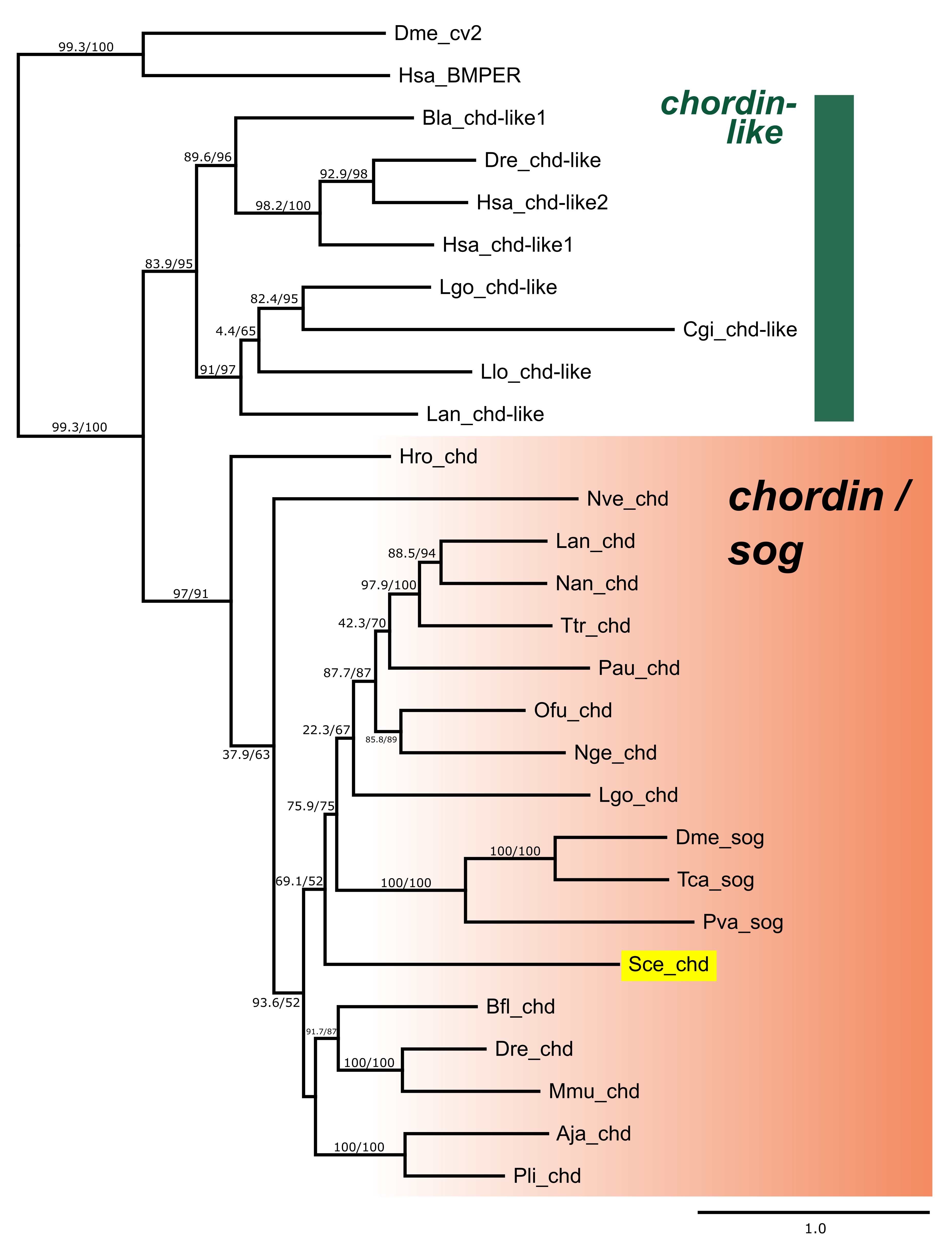


**Supplementary Figure 2.** Maximum Likelihood phylogenetic analysis of Chordin/Short gastrulation (Sog) amino acid sequences including deduced Chd/Sog protein from *Spadella cephaloptera* (highlighted in yellow). The tree was inferred using IQ-TREE based on bilaterian protein sequences obtained from published sources and NCBI GenBank BLAST searches. Branch support values are shown as SH-aLRT (%) / ultrafast bootstrap support (UFBS %). Support labels are omitted for nodes where both values are <50. Species abbreviations are provided in Supplementary Table 1.


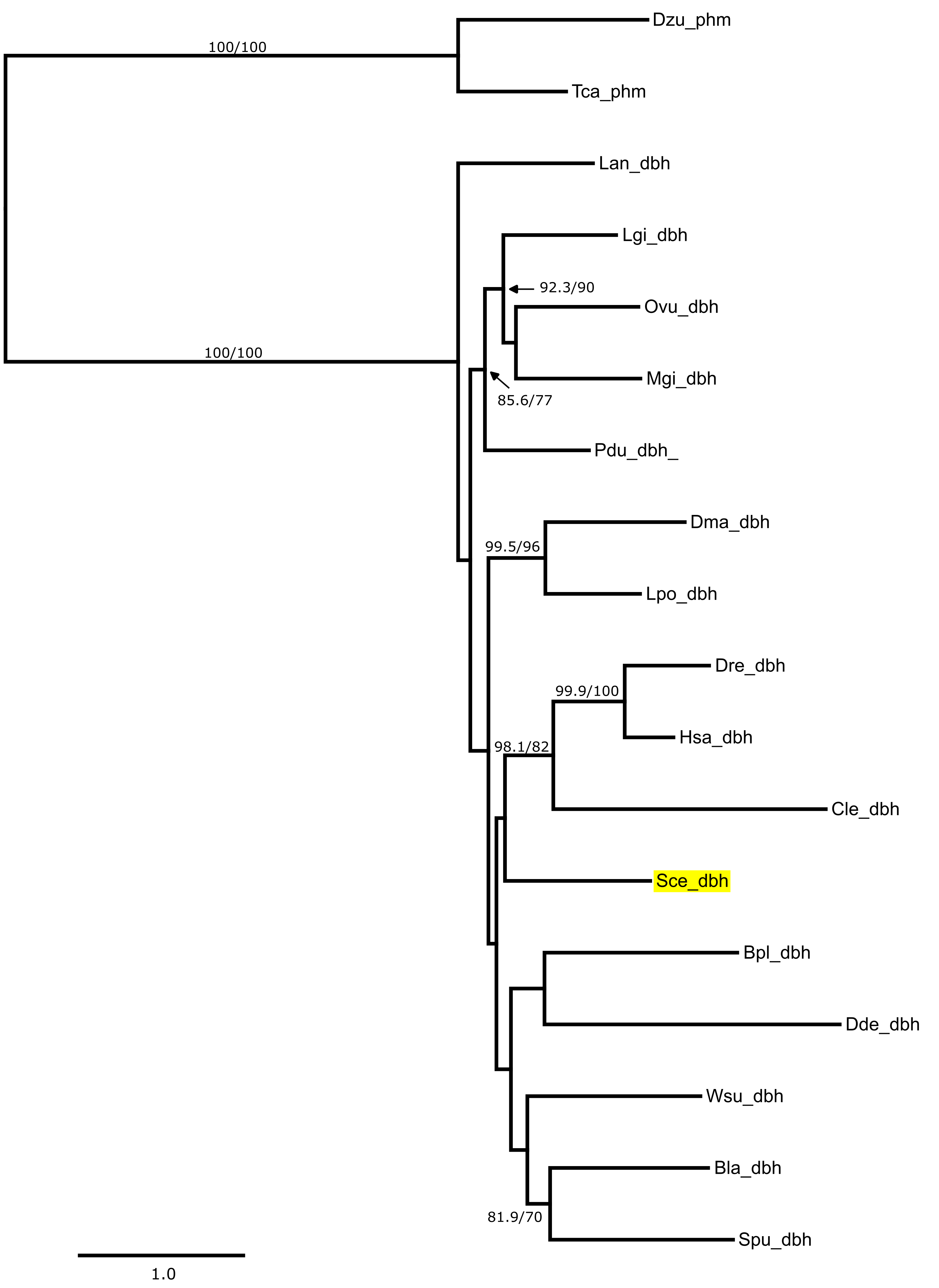


**Supplementary Figure 3.** Maximum Likelihood phylogenetic analysis of Dopamine-beta-hydroxylase (Dbh) amino acid sequences including deduced Dbh protein from *Spadella cephaloptera* (highlighted in yellow). The tree was inferred using IQ-TREE based on bilaterian protein sequences obtained from published sources and NCBI GenBank BLAST searches. Branch support values are shown as SH-aLRT (%) / ultrafast bootstrap support (UFBS %). Support labels are omitted for nodes where both values are <50. Species abbreviations are provided in Supplementary Table 1.


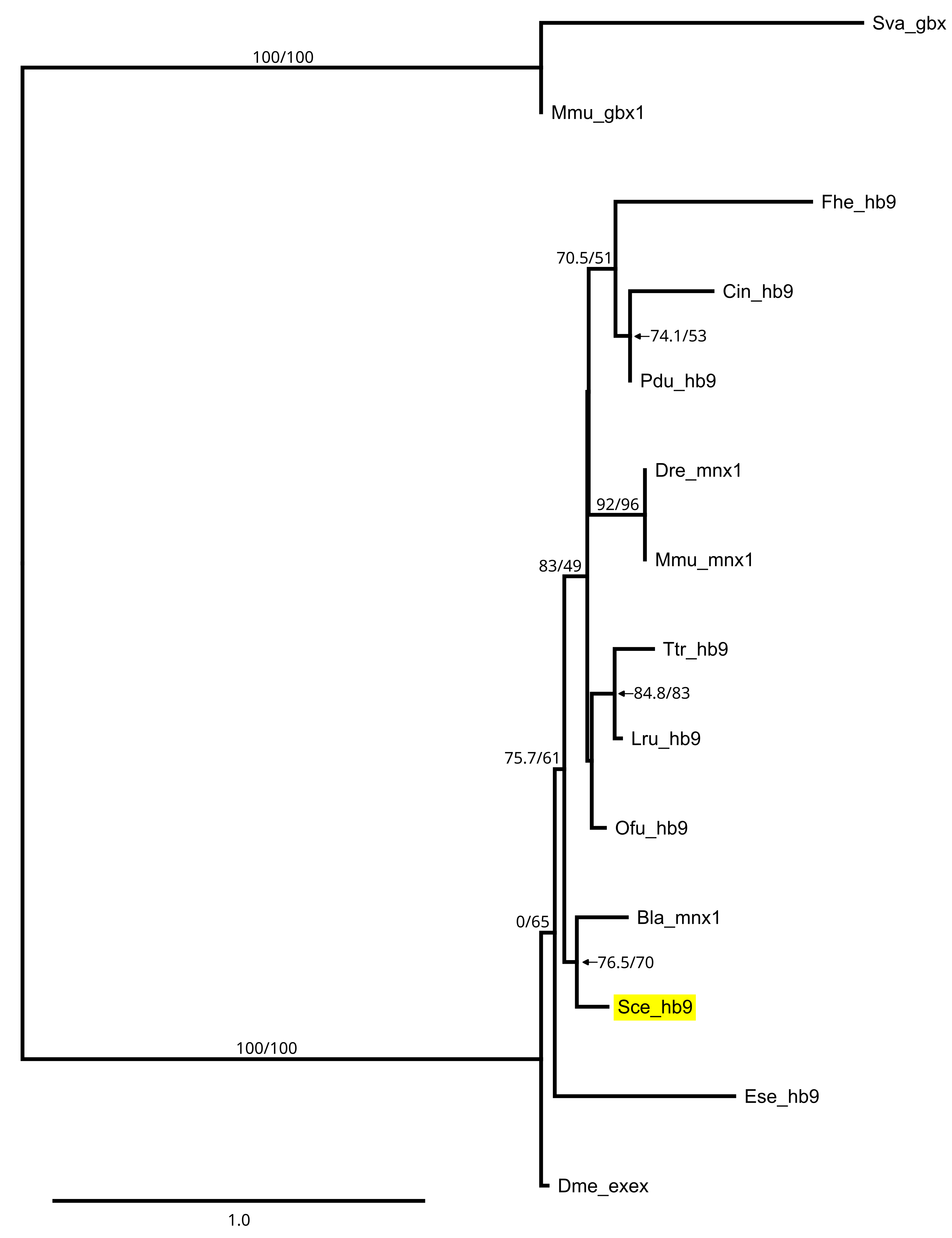


**Supplementary Figure 4.** Maximum Likelihood phylogenetic analysis of Hb9/Motor neuron and pancreas homeobox 1 (Mnx1)/Extra-extra (ex-ex) amino acid sequences including deduced Hb9/Mnx1/Exex protein from *Spadella cephaloptera* (highlighted in yellow). The tree was inferred using IQ-TREE based on bilaterian protein sequences obtained from published sources and NCBI GenBank BLAST searches. Branch support values are shown as SH-aLRT (%) / ultrafast bootstrap support (UFBS %). Support labels are omitted for nodes where both values are <50. Species abbreviations are provided in Supplementary Table 1.


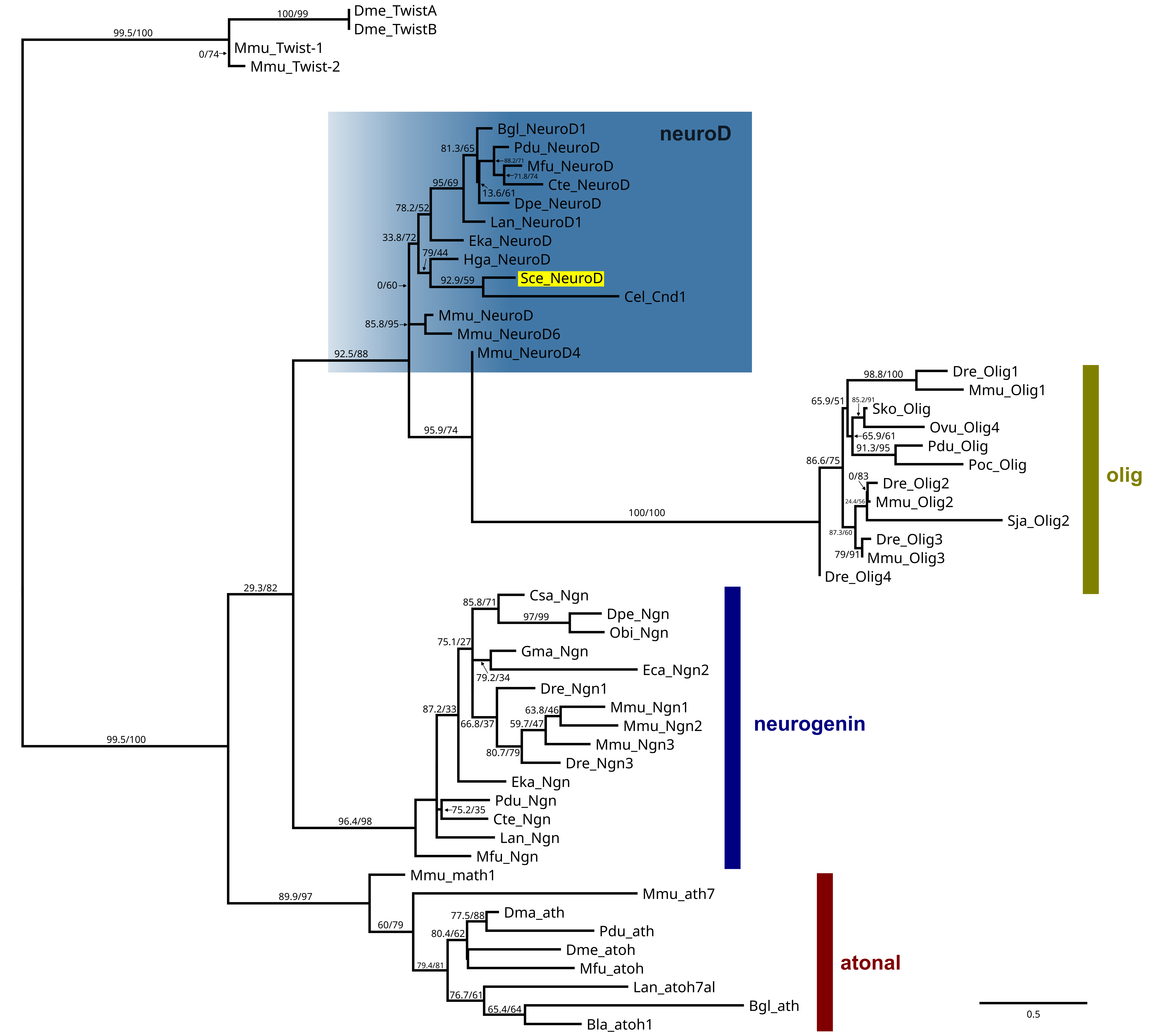


**Supplementary Figure 5.** Maximum Likelihood phylogenetic analysis of Neuronal differentiation 1 (NeuroD) amino acid sequences, including related bHLH family representatives and deduced NeuroD protein from *Spadella cephaloptera* (highlighted in yellow). The tree was inferred using IQ-TREE based on bilaterian protein sequences obtained from published sources and NCBI GenBank BLAST searches. Branch support values are shown as SH-aLRT (%) / ultrafast bootstrap support (UFBS %). Support labels are omitted for nodes where both values are <50. Species abbreviations are provided in Supplementary Table 1.





**Supplementary Figure 6.** Maximum Likelihood phylogenetic analysis of Nk6/Hgtx and related NK homeobox family amino acid sequences, including deduced Nk6/Hgtx protein from *Spadella cephaloptera* (highlighted in yellow). The tree was inferred using IQ-TREE based on bilaterian protein sequences obtained from published sources and NCBI GenBank BLAST searches. Branch support values are shown as SH-aLRT (%) / ultrafast bootstrap support (UFBS %). Support labels are omitted for nodes where both values are <50. Species abbreviations are provided in Supplementary Table 1.

**

**

**Supplementary Figure 7.** Maximum Likelihood phylogenetic analysis of SRY-related HMG box (Sox) amino acid sequences, including deduced protein SoxB proteins from *Spadella cephaloptera* (highlighted in yellow). The unrooted tree was inferred using IQ-TREE based on bilaterian protein sequences obtained from published sources and NCBI GenBank BLAST searches. Branch support values are shown as SH-aLRT (%) / ultrafast bootstrap support (UFBS %). SoxB1 and SoxB2 clades are highlighted in dark teal and light blue, respectively. Support labels are omitted for nodes where both values are <50. Species abbreviations are provided in Supplementary Table 1.


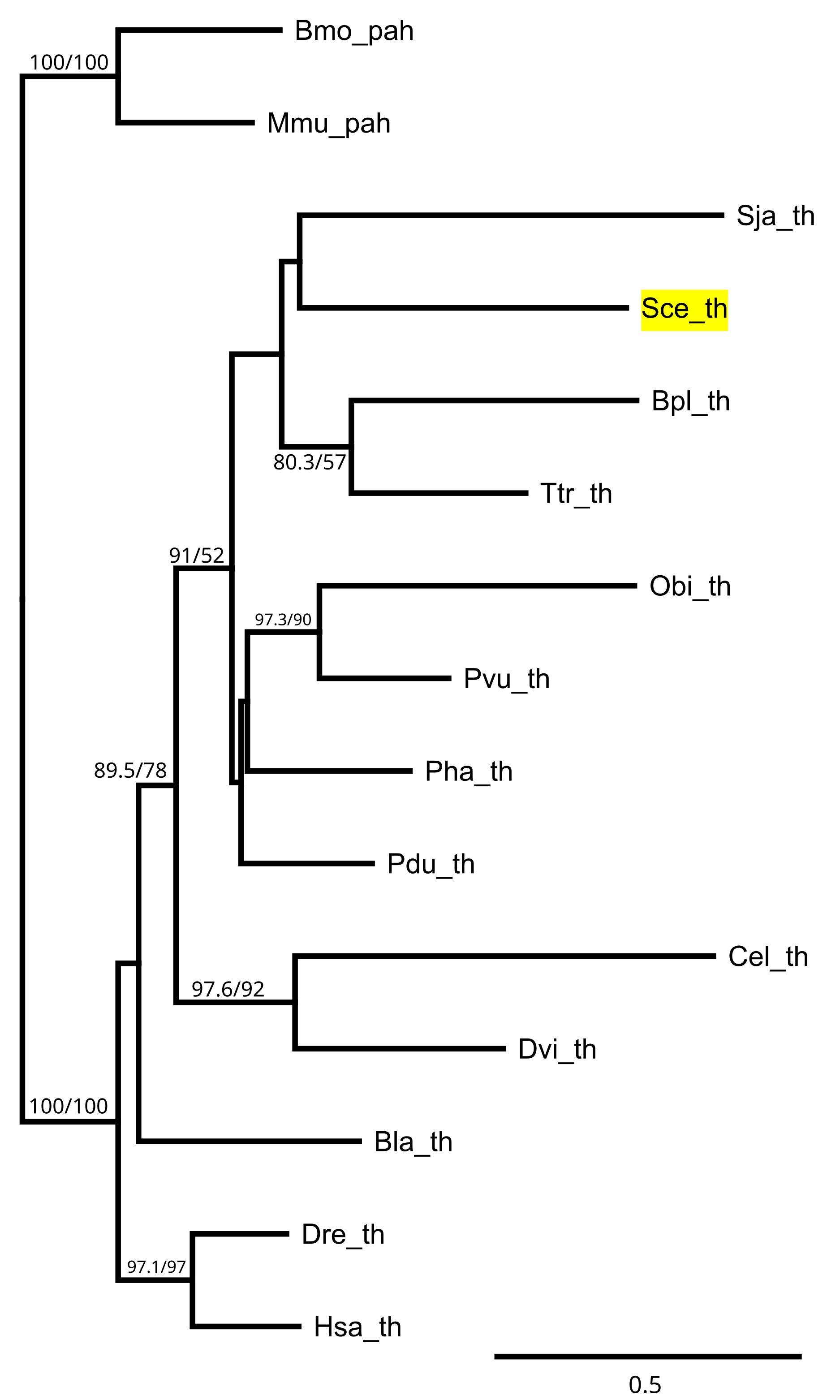


**Supplementary Figure 8.** Maximum Likelihood phylogenetic analysis of Tyrosine Hydroxylase (Th) amino acid sequences including deduced Th protein from *Spadella cephaloptera* (highlighted in yellow). The tree was inferred using IQ-TREE based on bilaterian protein sequences obtained from published sources and NCBI GenBank BLAST searches. Branch support values are shown as SH-aLRT (%) / ultrafast bootstrap support (UFBS %). Support labels are omitted for nodes where both values are <50. Species abbreviations are provided in Supplementary Table 1.

**
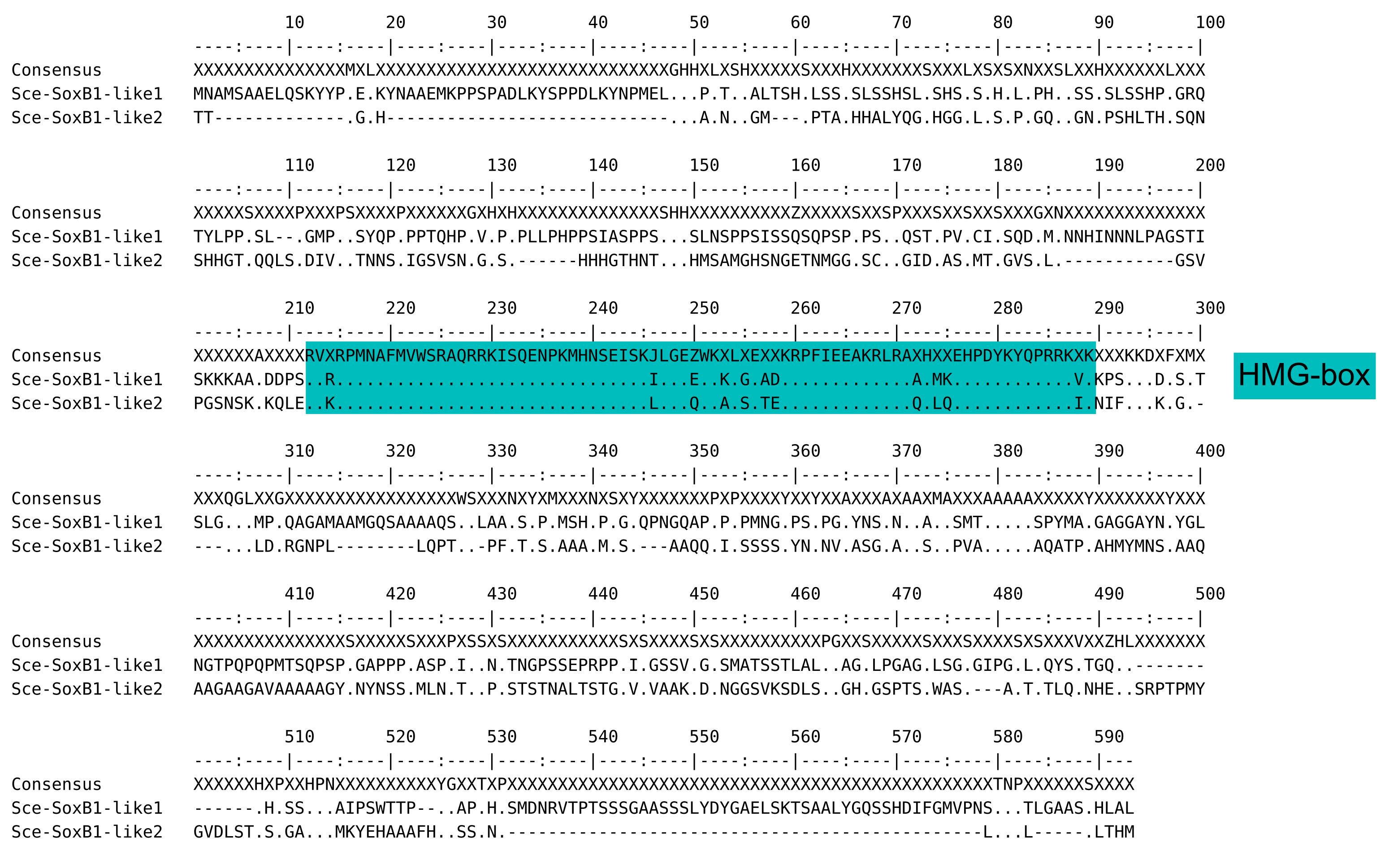
**

**Supplementary Figure 9.** Pairwise protein alignment of *Sce-SoxB1-like1* and *Sce-SoxB1-like2*. The HMG box is highlighted in teal. *Sce-SoxB1-like2* is 5′-partial relative to the complete *Sce-SoxB1-like1* ORF. The full alignment file is provided in Supplementary Data.


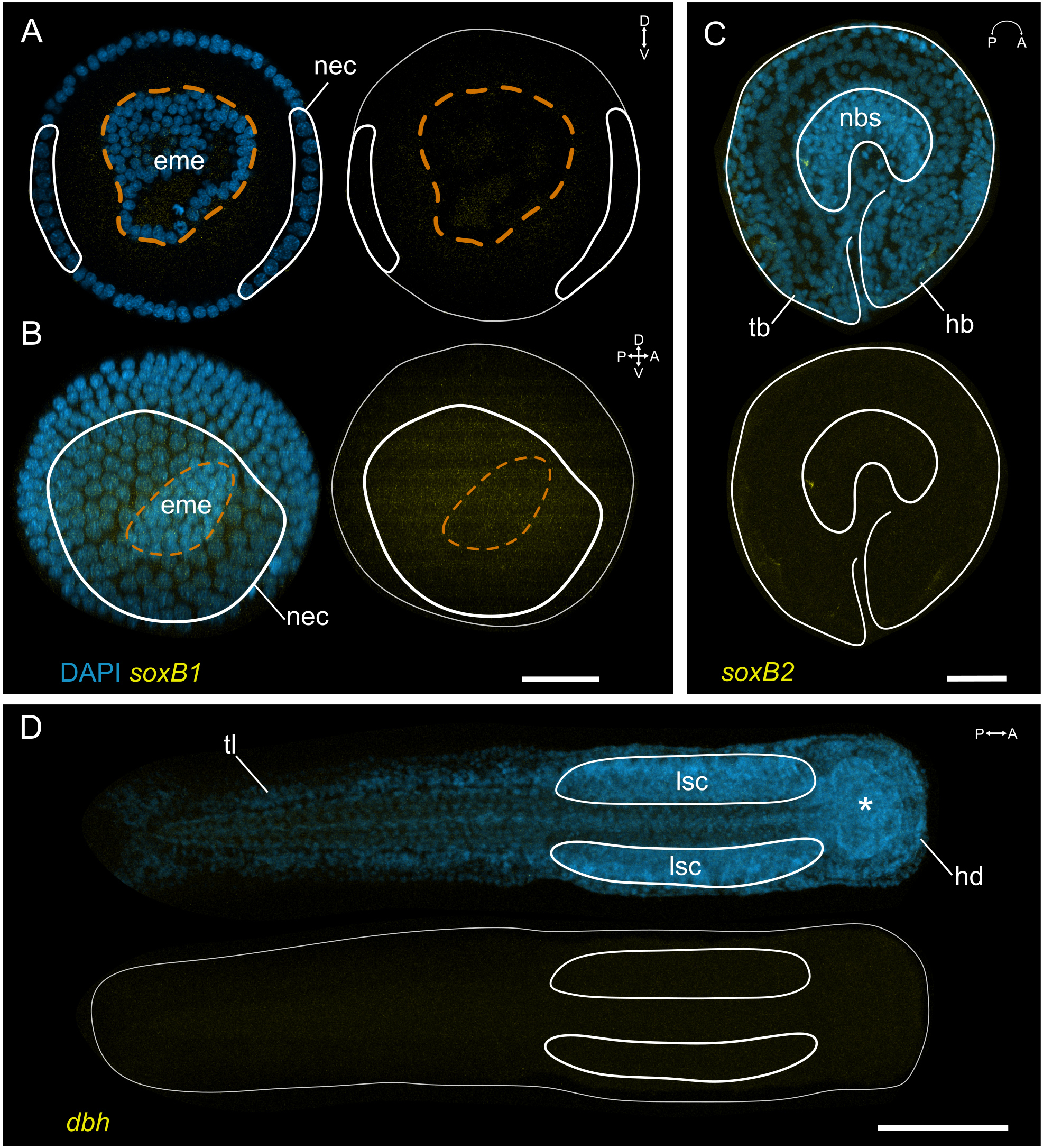


**Supplementary Figure 10.** Negative expression results for selected genes/stages.
(A, B) *Sce-soxB1* was not detected at early gastrula, shown in transverse view (A) and lateral view (B). In (B), the endomesoderm (orange outline) is partially visible due to the partial z-projection. (C) *Sce-soxB2* was not detected at early elongation, lateral view.
(D) *Sce-dbh* was not detected at hatchling stage. Asterisk marks the position of the mouth.

Scale bars: 50 µm, except (D): 100 µm. Orientation is indicated in the schematic representations and, where applicable, in the upper right corner of each panel. Abbreviations: eme, endomesoderm; epi, epidermis; hb, head bud; hd, head; nbs, neural cells of the developing VNC; nec, neuroectoderm; tb, tail bud; tl, tail.


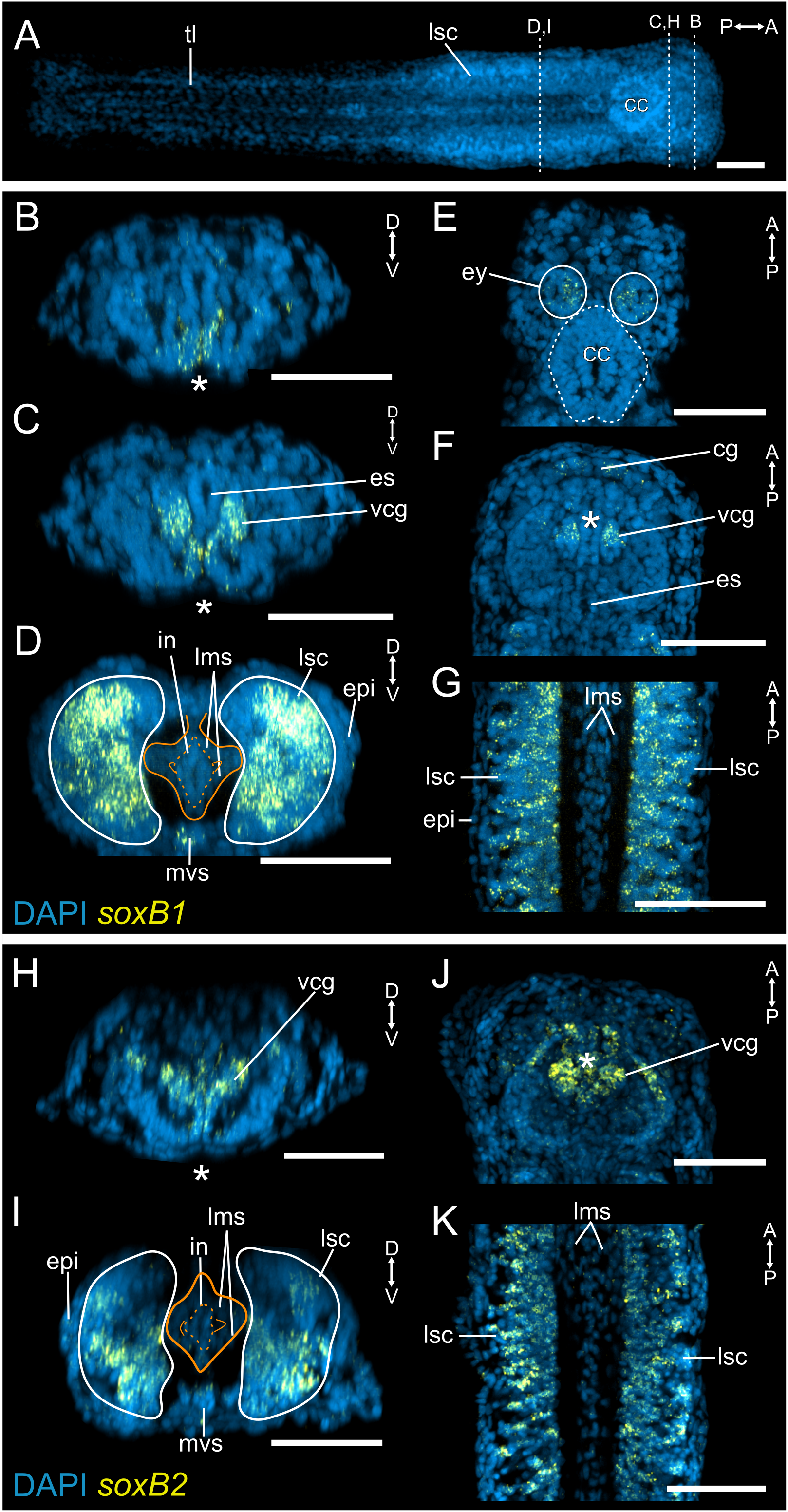


## **Supplementary Figure 11.** Expression patterns of *Sce-soxB1* and *Sce-soxB2* in the *Spadella cephaloptera* hatchling.

(A) Dorsal maximum projection of a DAPI-counterstained hatchling indicating the positions along the anterior–posterior axis from which transverse sections were collected.

(B–G) *Sce-soxB1* expression. (B–D) Transverse sections through the head (B, C) and trunk (D). Orange outline demarcates the mesodermal derivatives, including the trunk longitudinal muscles. (E, F) Dorsal sections of the head. (E) dorsal-most section showing expression in the eyes. (F) mid-level section showing expression in the presumptive anteroventral cephalic ganglion anlage. (G) Mid-dorsal section of the trunk.

(H–J) *Sce-soxB2* expression. (H, I) Transverse sections through the head (H) and trunk (I). (J) Mid-dorsal sections of the head and trunk.

Graphic annotations: White outlines demarcate the lateral somata clusters; orange outlines indicate mesodermal cells and the intestinal region. The asterisk marks the position of the mouth.

Scale bars: 50 µm, except (A): 100 µm. Asterisks indicate the position of the future mouth opening. Orientation is indicated in the upper right corner of each panel. Abbreviations: cc, corona ciliata; epi, epidermis; ey, eye; in, intestine; lsc, lateral somata clusters; lms, longitudinal muscle somata; mvs, medioventral somata clusters; vcg, presumptive anteroventral cephalic ganglion anlage; tl, tail.

**
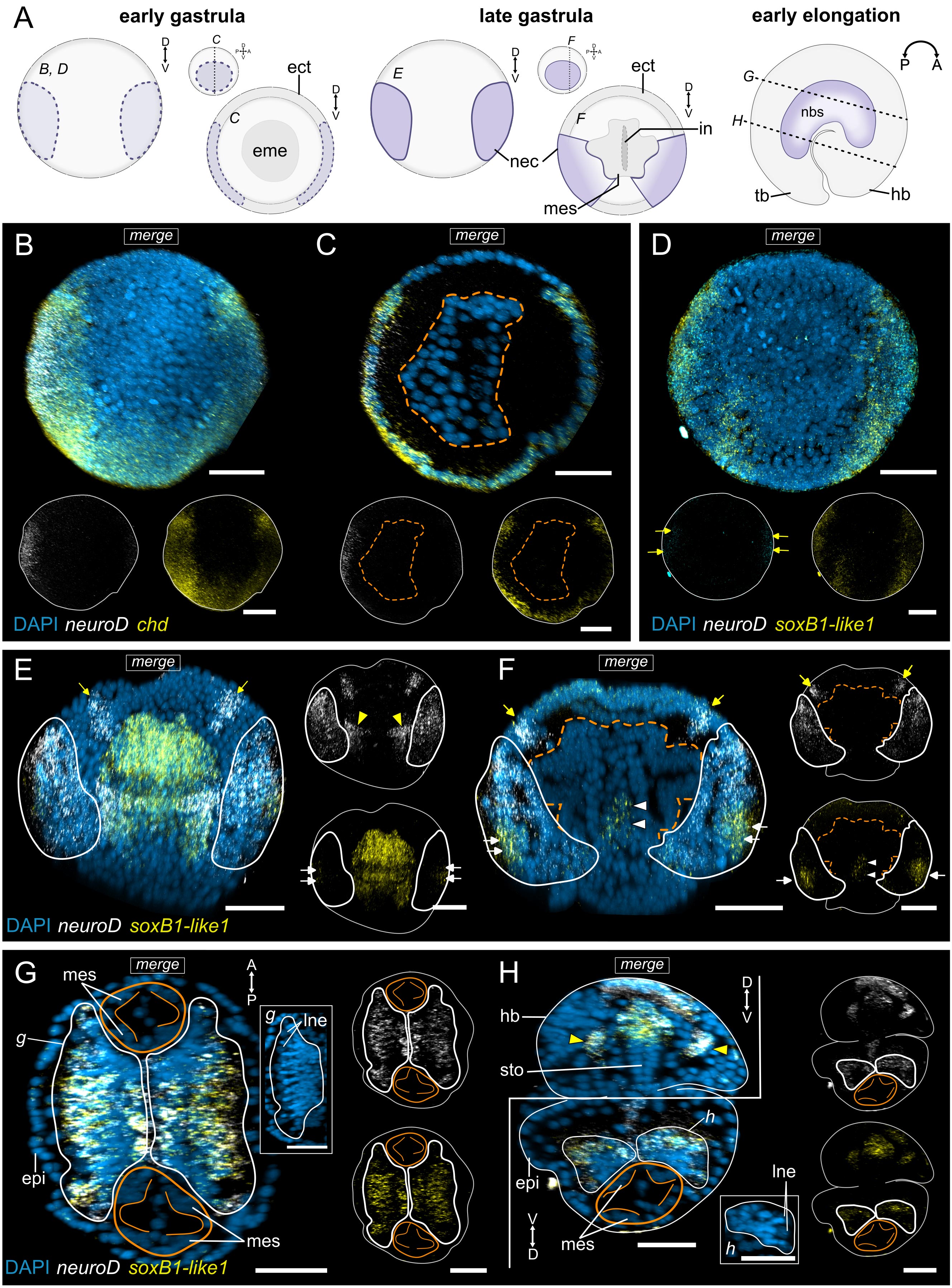
**

## **Supplementary Figure 12.** Double-label expression analyses of *Sce-neuroD* with *Sce-soxB1-like1* and *Sce-chd* during embryonic development of *Spadella cephaloptera*. Fluorescence panels are shown as a composite overlay (DAPI and two probe signals) together with the corresponding individual probe channels.

(A) Schematic representations of early gastrula, late gastrula, and early elongation stages. Early and late gastrula stages are shown in transverse views (full view and transverse section), while the early elongation stage is shown in lateral view. Neurogenic regions, including the NEC and neural cells of the nascent VNC, are indicated in purple.

(B–D) Early gastrula. (B) Transverse maximum projection and (C) transverse section showing *Sce-neuroD* and *Sce-chordin* expression. Orange dashed outline demarcates the endomesoderm. (D) Transverse maximum projection showing *Sce-neuroD* and *Sce-soxB1-like1* expression. Separate channels are shown without DAPI to improve visualization. Yellow arrows indicate *Sce-neuroD* expression domains.

(E, F) Late gastrula. (E) Transverse maximum projection and (F) transverse section. Yellow arrows indicate *Sce-neuroD* expression detected outside the anatomically defined NEC; yellow arrowheads highlight paired anterior ectodermal expression domains; white arrows mark the *Sce-soxB1-like1* expression in the ventral NEC; white arrowheads marks *soxB1-like1* expression in the anterior endomesoderm

(G, H) Early elongation. (G) Dorsal and (H) transverse section through the early elongation trunk. Orange outlines demarcate the mesodermal cells. Yellow arrows highlight paired *Sce-neuroD*⁺/*Sce-soxB1-like1*⁺ expression domains in the head bud. Insets *g* and *h* show the corresponding DAPI-only views of the left portion of the nascent VNC.

Graphic annotations: White outlines indicate neural territories; orange outlines indicate the endomesoderm in gastrula stages and mesodermal cells and the intestinal region in the early elongation stage.

Scale bars: 50 µm. Orientation is indicated in the schematic representations and, where applicable, in the upper right corner of each panel. Abbreviations: ect, ectoderm; eme, endomesoderm; epi, epidermis; hb, head bud; in, intestine; lne, large-nucleus neuroectodermal cells; mes, mesodermal cells; nbs, neural cells of the developing VNC; nec, neuroectoderm; sto, stomodeum; tb, tail bud.

**
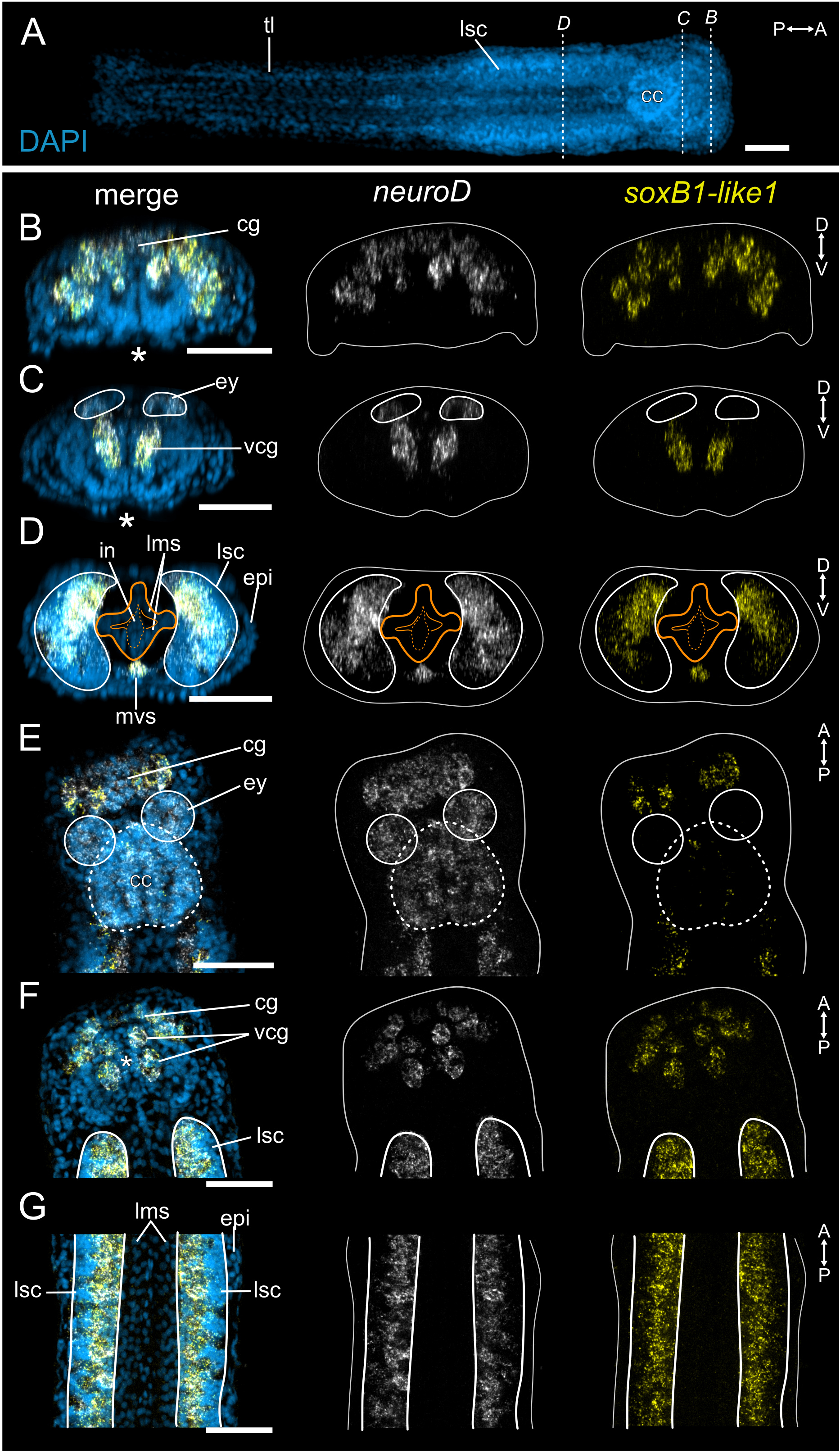
**

## **Supplementary Figure 13.** Double-label expression analyses of *Sce-neuroD* and *Sce-soxB1-like1* in the *Spadella cephaloptera* hatchling. Fluorescence panels are shown as a composite overlay (DAPI and two probe signals) together with the corresponding individual probe channels.

(A) Dorsal maximum projection of a DAPI-counterstained hatchling indicating the positions along the anterior–posterior axis from which transverse sections were collected.

(B–G) Expression shown as merged and separate channels. (B–D) Transverse sections through the head (B, C) and trunk (D). Orange outline demarcates the mesodermal derivatives, including the trunk longitudinal muscles, and the intestine (dashed). The asterisk marks the position of the mouth. (E, F) Dorsal sections of the head. (E) Dorsal-most section showing expression of *Sce-neuroD* in the eyes and corona ciliata. (F) Mid-level section showing expression in the presumptive anteroventral cephalic ganglion anlage and cephalic ganglion. (G) Mid-dorsal section of the trunk.

Scale bars: 50 µm, except (A): 100 µm. Asterisks indicate the position of the future mouth opening. Orientation is indicated in the upper right corner of each panel. Abbreviations: cc, corona ciliata; cg, cerebral ganglion; epi, epidermis; ey, eye; in, intestine; lsc, lateral somata clusters; lms, longitudinal muscle somata; mvs, medioventral somata clusters; vcg, presumptive anteroventral cephalic ganglion anlage; tl, tail.

**
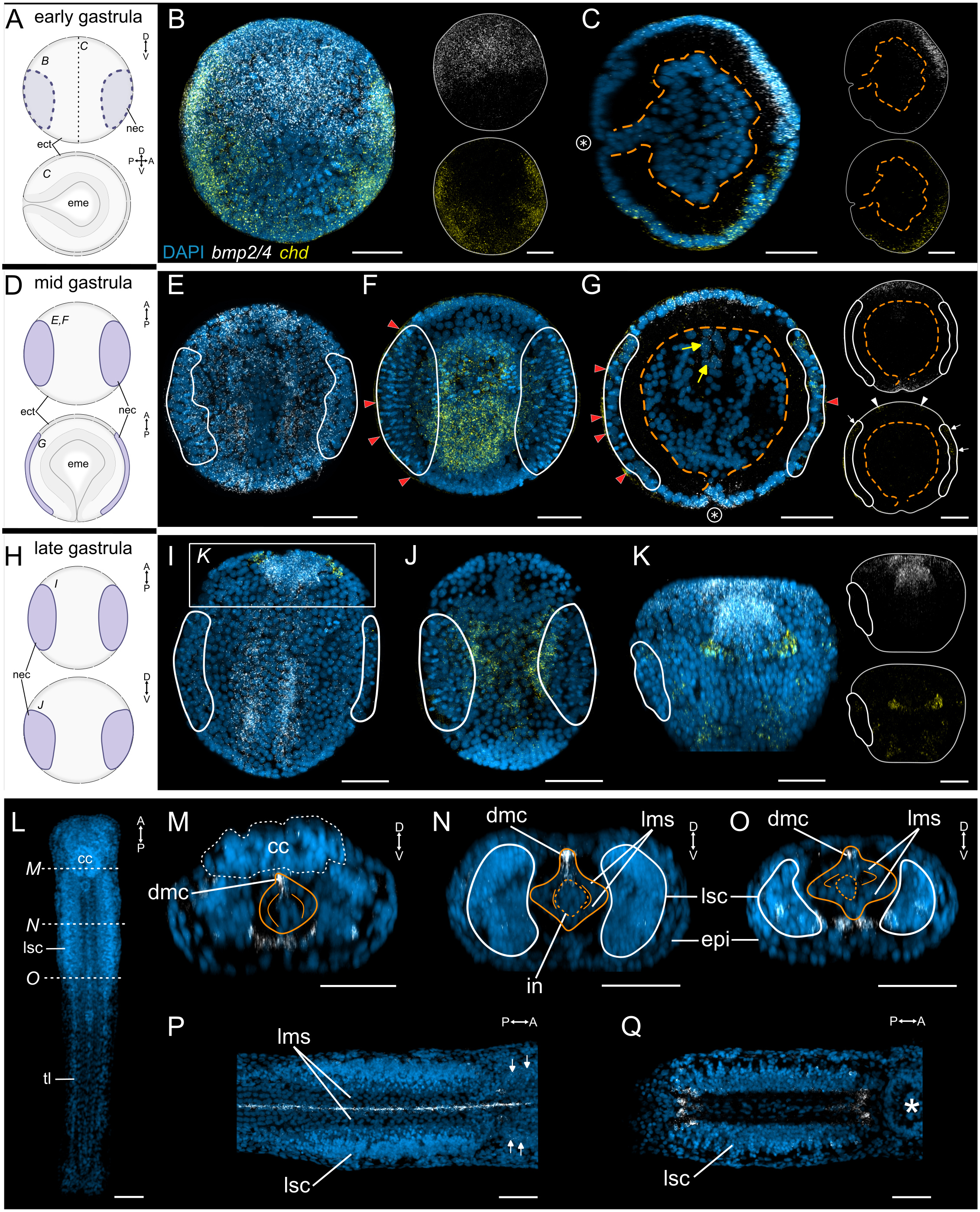
**

## **Supplementary Figure 14.** Double-label expression analyses of *Sce-bmp2/4* and *Sce-chd* during embryonic development and *Sce-bmp2/4* expression in the hatchling of *Spadella cephaloptera*. Where applicable, fluorescence panels are shown as a composite overlay (DAPI and two probe signals) together with the corresponding individual probe channels.

(A, D, H) Schematic representations of early (A), mid (D), and late (H) gastrula stages. The early gastrula is shown in transverse view (top) and as a lateral section (bottom); the mid gastrula is shown in full dorsal view (top) and as a dorsal section (bottom); the late gastrula is shown in full dorsal view (top) and full transverse view (bottom). The NEC is indicated in purple.

(B, C) Early gastrula. (B) Transverse maximum projection and (C) lateral section through the blastoporal region showing merged channels (left) and individual channels (right). Dashed outlines demarcate the endomesoderm. Encircled asterisks mark the blastoporal region.

(E–G) Mid gastrula. (E, F) Dorsal maximum projections of the dorsal (E) and ventral (F) portions of the embryo. White outlines demarcate the NEC. Red arrowheads indicate non-specific signal from the inner shell layer. (G) Dorsal section through the blastoporal region showing *Sce-bmp2/4* expression. Yellow arrows mark *Sce-bmp2/4* expression in a subset of endomesodermal cells. White arrows indicate *Sce-chd* expression in NEC cells, and white arrowheads highlight anterior ectodermal *Sce-chd* expression.

(I–K) Late gastrula. (I, K) Dorsal maximum projections of the dorsal (I) and ventral (J) portions of the embryo. (K) Transverse maximum projection of the anterior region.

(L–Q) *Sce-bmp2/4* expression in the hatchling. (L) Dorsal maximum projection of a DAPI-counterstained hatchling indicating the positions along the anterior–posterior axis from which transverse sections were collected. (M–O) Transverse sections through the head (M) and trunk (N, O). Orange outlines demarcate mesodermal derivatives, including dorsal medial cells and trunk longitudinal muscles, and the intestine (dashed outline). (P, Q) Dorsal sections of the trunk. (P) Mid-dorsal section showing expression in the dorsal medial cells (yellow arrows) along the trunk and weak signal near the grasping spine region (white arrows). (Q) Ventral section showing expression in cells near the head–trunk and trunk–tail boundaries. The asterisk indicate the position of the mouth.

Graphic annotations: White outlines indicate neural territories; orange outlines indicate the endomesoderm in gastrula stages and mesodermal cells and the intestinal region in the hatchling.

Scale bars: 50 µm; except (L): 100 µm. Orientation is indicated in the schematic representations and, where applicable, in the upper right corner of each panel. Abbreviations: cc, corona ciliata; ect, ectoderm; eme, endomesoderm; epi, epidermis; dmc, dorsal medial cells; in, intestine; lms, longitudinal muscle somata; nec, neuroectoderm; tl, tail.


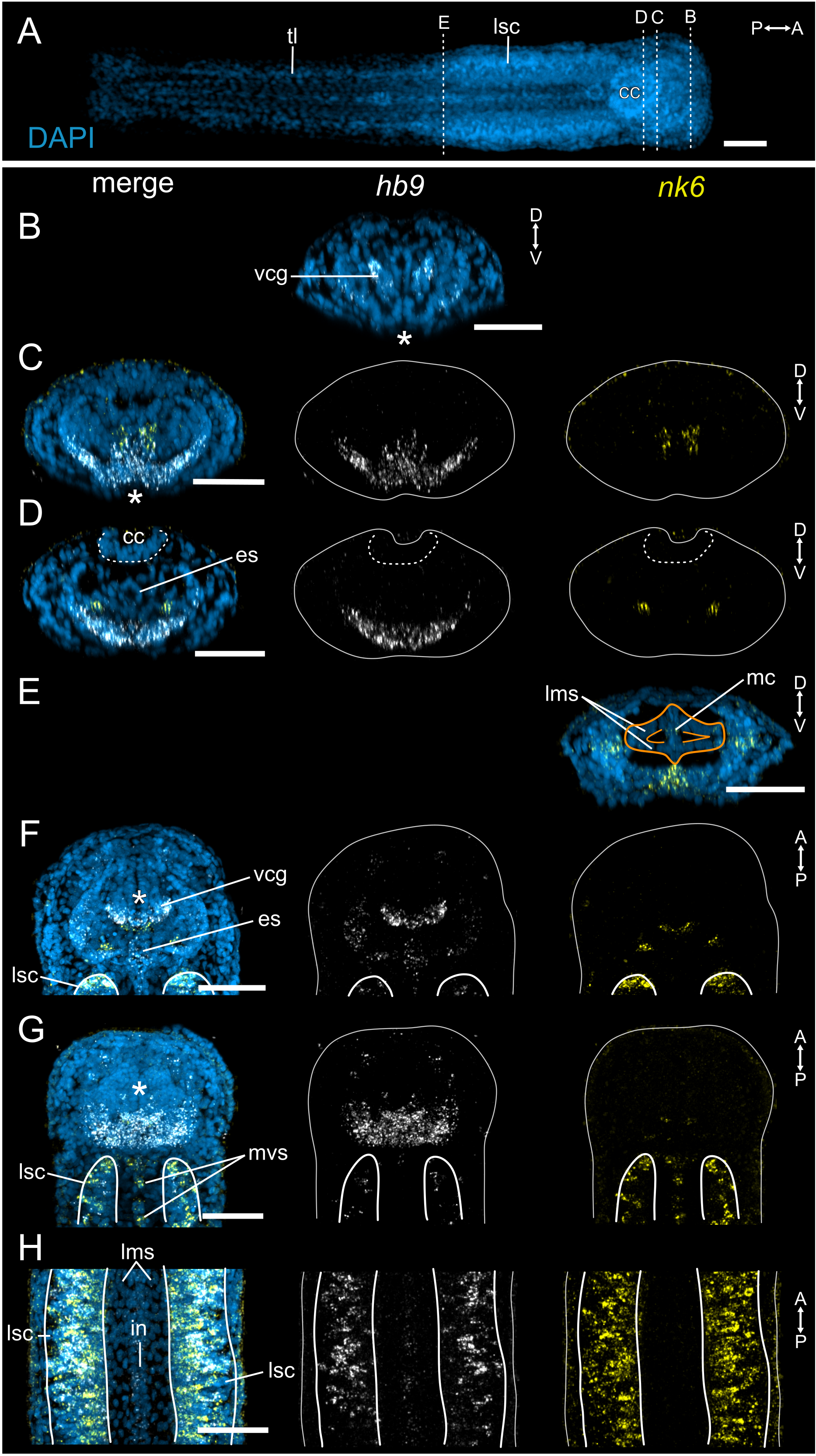


## **Supplementary Figure 15.** Double-label expression analyses of *Sce-hb9* and *Sce-nk6* in the *Spadella cephaloptera* hatchling. Where applicable, fluorescence panels are shown as a composite overlay (DAPI and two probe signals) together with the corresponding individual probe channels.

(A) Dorsal maximum projection of a DAPI-counterstained hatchling indicating the positions along the anterior–posterior axis from which transverse sections were collected.

(B–H) Expression shown as merged images and separate channels.
(B–E) Transverse sections through the head (B–D) and trunk–tail boundary (E). *Sce-hb9* is broadly expressed in ventral head cells corresponding to the presumptive perioral epidermis (B–D), whereas *Sce-nk6* expression is detected in discrete cells of the head (C, D) and posterior trunk (E). The asterisk marks the position of the mouth. Orange outlines demarcate mesodermal derivatives, including mesenterial cells and trunk longitudinal muscles.

(F–H) Dorsal sections. (F) Mid-dorsal section of the head showing expression in the presumptive anteroventral cephalic ganglion anlage and esophagus. (G) Ventral-most section of the head showing *Sce-nk6* expression in the medioventral somata clusters. (H) Mid-dorsal section of the trunk. White outlines demarcate the lateral somata clusters.

Scale bars: 50 µm, except (A): 100 µm. Asterisks indicate the position of the future mouth opening. Orientation is indicated in the upper right corner of each panel. Abbreviations: cc, corona ciliata; epi, epidermis; es, esophagus; in, intestine; lsc, lateral somata clusters; lms, longitudinal muscle somata; mc, mesenterial cells; mvs, medioventral somata clusters; vcg, presumptive anteroventral cephalic ganglion anlage.

**
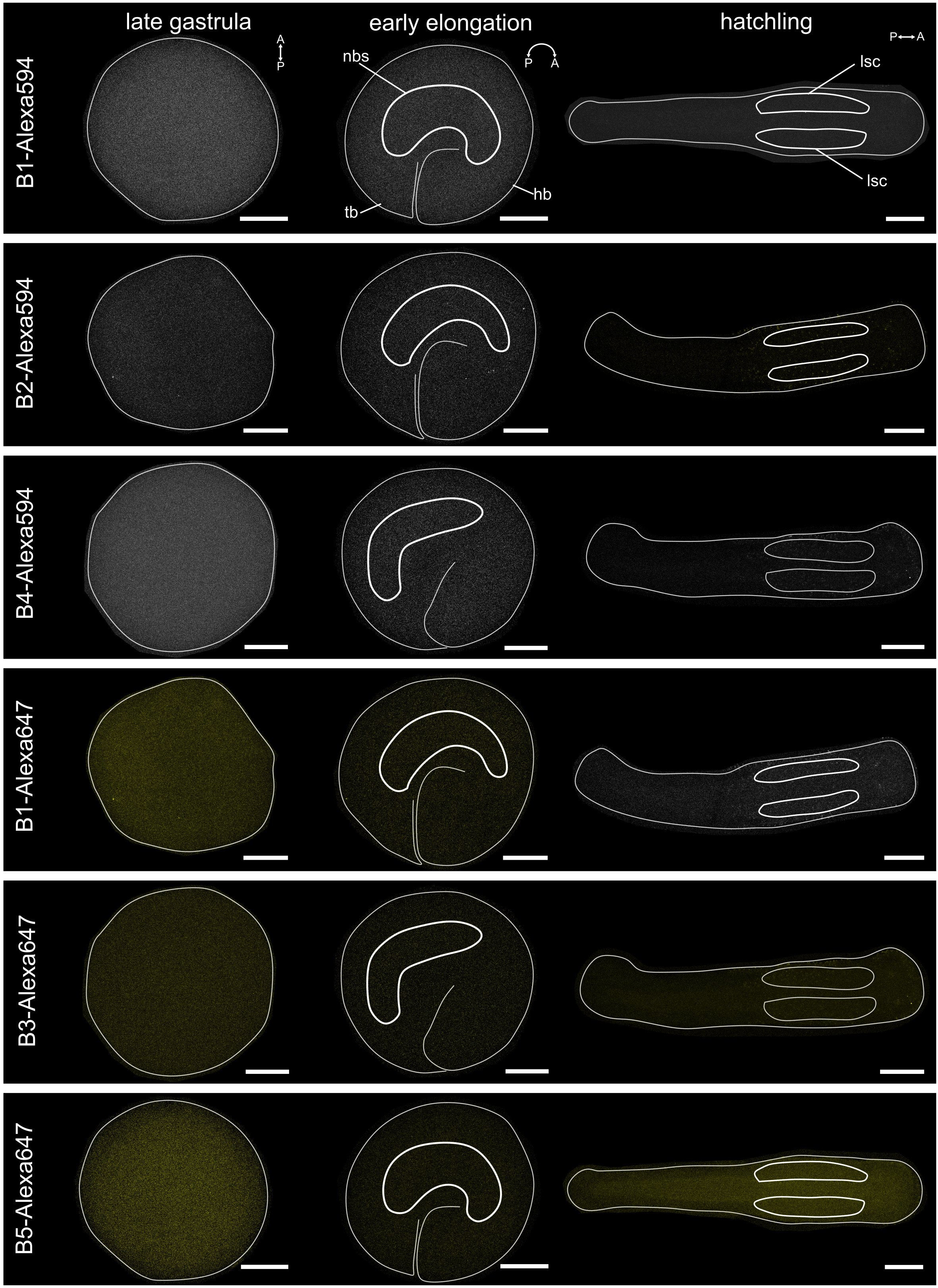
**

**Supplementary Figure 16.** Negative controls for HCR hairpin–fluorophore combinations. Negative control samples were processed using hairpins only, without probe incubation, to assess background signal under the same hairpin/fluorophore and imaging conditions used for the experimental specimens. The hairpin/laser combinations shown correspond to those used in the main dataset. The hairpin–laser combination is indicated at the left of each row, and the developmental stage at the top of each column. Shown are early gastrula (full-stack lateral view), late gastrula (full-stack dorsal view), and hatchling (full-stack dorsal view). Orientation is indicated in the upper right of each panel.
Scale bars: 50 µm; except hatchlings: 100 µm.

Abbreviations: hb, head bud; lsc, lateral somata clusters; in, intestine; nbs, neural cells of the developing VNC; tb, tail bud.
